# QM/MM Study of Partial Dissociation of S2B for the E_2_ Intermediate of Nitrogenase

**DOI:** 10.1021/acs.inorgchem.2c02488

**Published:** 2022-10-28

**Authors:** Hao Jiang, Oskar K. G. Svensson, Ulf Ryde

**Affiliations:** Department of Theoretical Chemistry, Lund University, Chemical Centre, SE-221 00Lund, Sweden

## Abstract

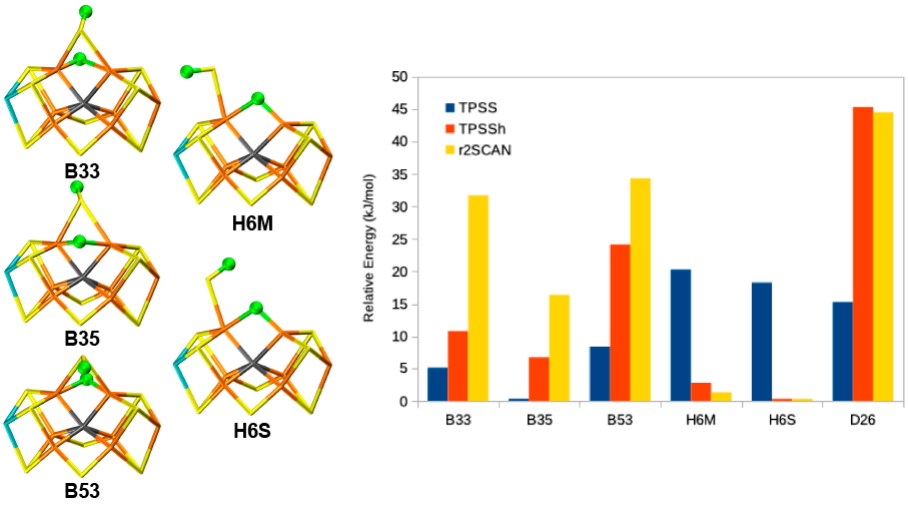

Nitrogenase is the only enzyme that can cleave the triple bond in N_2_, making nitrogen available for all lifeforms. Previous computational studies have given widely diverging results regarding the reaction mechanism of the enzyme. For example, some recent studies have suggested that one of the μ_2_-bridging sulfide ligands (S2B) may dissociate from one of the Fe ions when protonated in the doubly reduced and protonated E_2_ state, whereas other studies indicated that such half-dissociated states are unfavorable. We have examined how the relative energies of 26 structures of the E_2_ state depend on details of combined quantum mechanical and molecular mechanical (QM/MM) calculations. We show that the selection of the broken-symmetry state, the basis set, relativistic effects, the size of the QM system, relaxation of the surroundings, and the conformations of the bound protons may affect the relative energies of the various structures by up to 12, 22, 9, 20, 37, and 33 kJ/mol, respectively. However, they do not change the preferred type of structures. On the other hand, the choice of the DFT functional strongly affects the preferences. The hybrid B3LYP functional strongly prefers doubly protonation of the central carbide ion, but such a structure is not consistent with experimental EPR data. Other functionals suggest structures with a hydride ion, in agreement with the experiments, and show that the ion bridges between Fe2 and Fe6. Moreover, there are two structures of the same type that are degenerate within 1–5 kJ/mol, in agreement with the observation of two EPR signals. However, the pure generalized gradient approximation (GGA) functional TPSS favors structures with a protonated S2B also bridging Fe2 and Fe6, whereas r^2^SCAN (meta-GGA) and TPSSh (hybrid) prefer structures with S2B dissociated from Fe2 (but remaining bound to Fe6). The energy difference between the two types of structure is so small (7–18 kJ/mol) that both types need to be considered in future investigations of the mechanism of nitrogenase.

## Abstract 2

We have studied the relative stabilities of 26 structural models of the E_2_ state of Mo-nitrogenase with QM/MM calculations. The results depend strongly on the DFT method. B3LYP prefers a doubly protonated carbide ion, inconsistent with experimental data. Other methods suggest a hydride ion bridging Fe2 and Fe6, and a protonated S2B sulfide ion, which is either bridging the same Fe ions (TPSS) or bind only to Fe6 (TPSSh and r^2^SCAN).

## Introduction

**Figure 1:**
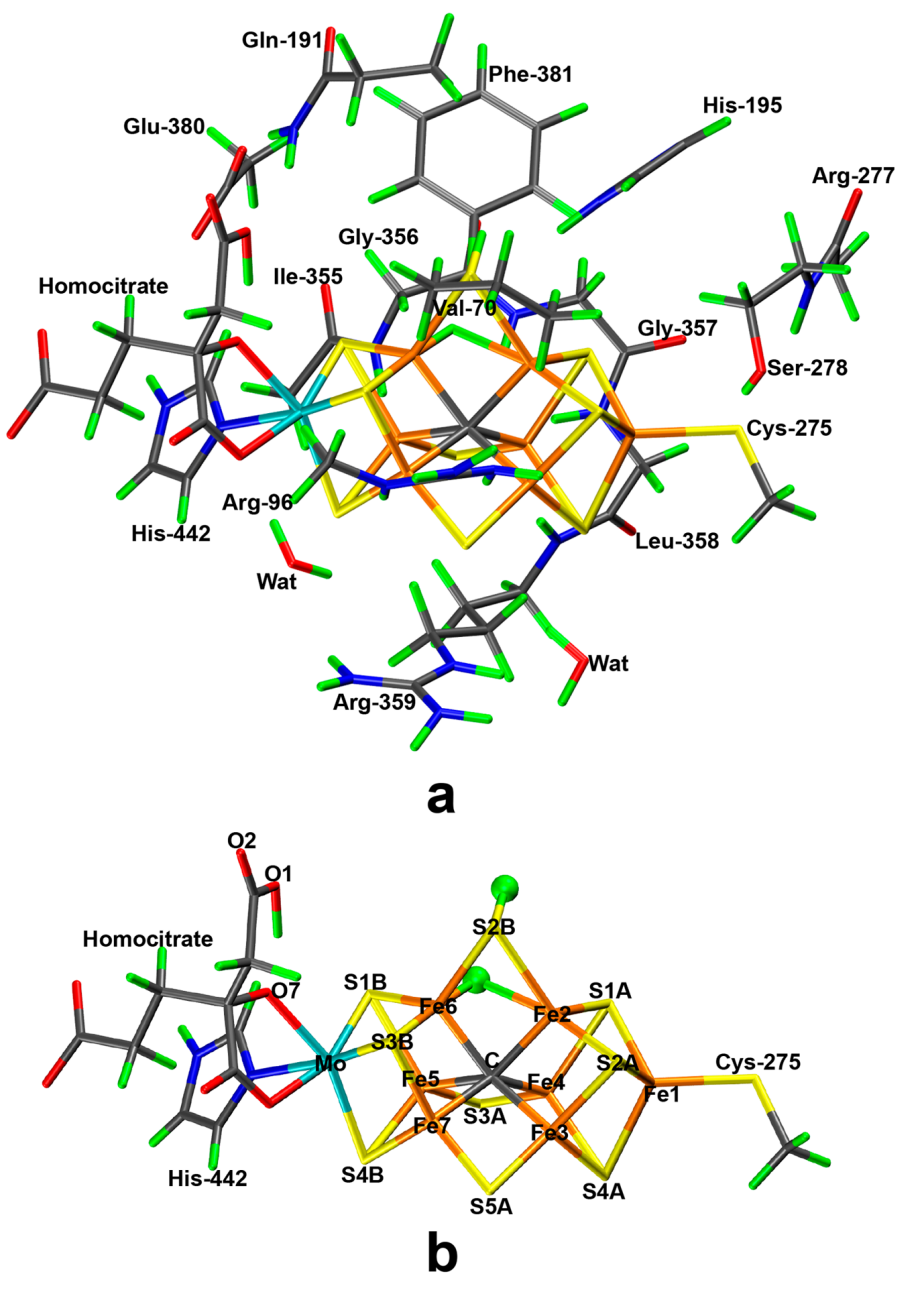
(a) Structure of the FeMo cluster (B33 structure), also illustrating the QM system used in the calculations, as well as nearby residues. (b) The FeMo cluster with atom names indicated.

Nitrogenases (EC 1.18/19.6.1) are the only group of enzymes that can cleave the inert triple bond in N_2_, making atmospheric nitrogen available for all lifeforms.^1−3^ Crystal structures have shown that the active site of Mo-nitrogenase is a complicated MoFe_7_S_9_C(homocitrate) cluster (the FeMo cluster), which is bound to the protein with one His and one Cys residue (Figure 1).^4−8^ Alternative nitrogenases also exist with the Mo ion replaced with either vanadium or iron, but they have lower activities toward N_2_.^9^

The nitrogenase reaction requires 16 ATP molecules to convert one N_2_ molecule to two NH_3_ molecules:^1−3^
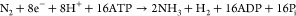
1

In addition, H_2_ seems to be a mandatory byproduct. The reaction consumes eight electrons and protons. It is normally assumed that each reduction of the cluster leads to the uptake of one proton. Therefore, the reaction is normally described by eight intermediates, E_0_–E_7_, differing in the number of added electrons and protons.^10^ Extensive biochemical, kinetic, and spectroscopic studies have indicated that the enzyme needs to be reduced four times (from E_0_ to E_4_) before N_2_ can bind through reductive elimination of H_2_.^1,2,11−17^ Electron nuclear double resonance (ENDOR) experiments suggest that the E_4_ state contains two hydride ions that bridge a pair of Fe ions each.^2,14,15^

Nitrogenase has also been extensively studied by computational methods.^18^ Unfortunately, they have suggested very disparate reaction mechanisms. In fact, they do not even agree on the structure of the key E_4_ state.^3,18−26^ Structures have been suggested with the central carbide ion triply protonated or various combinations of protonated sulfide and iron ions (both bridging and terminal). Even for structures with two bridging hydride ions, the suggested models differ in what iron ions are involved (typically Fe2/Fe6, Fe3/Fe7, or Fe 4/Fe5, but possibly the same pair for both hydrides; atom names are indicated in Figure 1b), the position of the hydride ion relative to the μ_2_-bridging sulfide ions, and whether the latter remains bound or not. An important reason for this discrepancy is that different DFT method give relative energies of different protonation states that can differ by over 600 kJ/mol, depending mainly on the amount of Hartree–Fock exchange in the method.^23^

A way to sort out these problems is to study simpler states with a lower number of possibilities. Most computational^18,27^ and experimental^28,29^ studies agree that the E_1_ state involves the protonation of the S2B μ_2_-bridging sulfide ligand of the FeMo cluster (but a recent experimental study of Fe-nitrogenase instead suggested a hydride ion^30^).

However, for the E_2_ state, the predictions of different DFT methods start to diverge. Pure generalized gradient approximation (GGA) functionals suggest that the second proton binds to a Fe ion.^27^ Thereby, it formally forms a hydride ion and brings the oxidation state of the cluster back to that of the resting E_0_ state, explaining why the same source of the electrons can be used for all E_*n*_ states.^31^ Experimentally, the E_2_ state has been studied by electron paramagnetic resonance (EPR) spectroscopy.^32−34^ Two signals are observed, which have been interpreted as structures that both contain a hydride ion bound to one or two Fe ions. The two structures are nearly degenerate (within 4–8 kJ/mol) and isomerization between them involves some structural relaxation of the surroundings of the FeMo cluster.

Recently, several groups have suggested that the protonated S2B ligand may dissociate from one of its two Fe ions (Fe2 or Fe6).^26,35−37^ In particular, Thorhallsson and Bjornsson (T&B) performed a study of the E_2_ state of the FeMo cluster with the TPSSh functional.^38^ They compared the relative energies of 18 different states, involving protonation of either the three μ_2_ bridging sulfide ions, the Fe ions, or the central carbide. With a QM/MM model, the most favorable structures had either the two protons on S2B and S5A, or a bridging hydride between Fe2 and Fe6 and a proton on S2B, which had dissociated from Fe2.

These results are quite different from what we obtained from a systematic study of ∼40 different protonation states of E_2_, all with a proton on S2B:^27^ With the TPSS functional, states with a bridging hydride ion between Fe2 and Fe6 (with the protonated S2B ligand still binding to both Fe2 and Fe6) were most stable and the two states with the hydride ion on either side of S2B differed by only 2 kJ/mol. However, a state with a terminal hydride ion on Fe5 was only 3 kJ/mol less stable. On the other hand, states with the second proton on S5A (pointing either toward S2B or S3A) were 30 and 37 kJ/mol less stable. No states with the protonated S2B dissociated from either Fe2 or Fe6 were observed, but they were not systematically explored.

Since half-dissociated S2B states have repeatedly been suggested to be involved in the reaction mechanism of nitrogenase,^26,35−37^ it is important to sort out whether these discrepancies in the computational predictions are caused by the QM model used, the DFT method or by other details in the calculations. Therefore, we here perform a systematic study of 26 different E_2_ structures at different levels of theory.

## Methods

### The Protein

The calculations were based on the 1.0-Å crystal structure of Mo nitrogenase from *Azotobacter vinelandii* (PDB code 3U7Q).^6^ The setup of the protein is identical with that of our previous studies.^22−24,39^ The entire heterotetramer was considered in the calculations, and the quantum mechanical (QM) calculations were concentrated on the FeMo clusters in the C subunit, because there is a buried imidazole molecule from the solvent rather close to the active site (∼11 Å) in the A subunit. The two P clusters and the FeMo cluster in subunit A were modeled by MM in the fully reduced and resting states, respectively, using a QM charge model.^22^ The protonation states of all residues were the same as before,^22^ and the homocitrate ligand was modeled in the singly protonated state with a proton shared between the hydroxyl group (O7 that coordinates to Mo) and the O1 carboxylate atom^22,40^ (all structures give two H–O distances of ∼1.1 and ∼1.4 Å; sometimes the proton is closer O1, sometimes O7, and when both structures can be found, they are typically degenerate within a few kJ/mol^22,27^). The protein was solvated in a sphere with a radius of 65 Å around the geometrical center of the protein. Cl^–^ and Na^+^ ions were added to an ionic strength of 0.2 M.^41^ The final system contained 133 915 atoms. For the protein, we used the Amber ff14SB force field,^42^ and water molecules were described by the TIP3P model.^43^ The metal sites^22,44^ were treated by a nonbonded model,^45^ and charges were obtained with the restrained electrostatic potential method.^46^

The FeMo cluster was modeled by MoFe_7_S_9_C(homocitrate)(CH_3_S)(imidazole), where the two last groups are models of Cys-275 and His-442. In addition, all groups that form hydrogen bonds to the FeMo cluster were also included in the QM model, viz. Arg-96, Gln-191 and His-195 (side chains), Ser-278 and Arg-359 (both backbone and side chain, including the CA and C and O atoms from Arg-277), Gly-356, Gly-357 and Leu-358 (backbone, including the CA and C and O atoms from Ile-355), as well as two water molecules. Finally, the side chain of Glu-380 was included because it forms hydrogen bonds to Gln191 and His-442, as well as the side chains of Val-70 and Phe-381 because they are close to S2B, Fe2 and Fe6, i.e., the prime binding sites of the two added protons or hydride ions. The QM system involved 191 atoms and is shown in Figure 1a. The net charge of QM region was −4 *e*. In one set of calculations, we instead used the same QM model as T&B,^38^ which contains 144 atoms and is shown in Figure S1 in the Supporting Information (the net charge is still −4 *e*).

### QM Calculations

All QM calculations were performed with the Turbomole software (version 7.5).^47^ All structures were studied with the TPSS,^48^ TPSSh,^49^ B3LYP,^50−52^ and r^2^SCAN^53^ functionals with the def2-SV(P) basis set.^54^ In one set of calculations, we instead used the def2-TZVPD basis set.^55^ The calculations were sped up by expanding the Coulomb interactions in an auxiliary basis set, the resolution-of-identity (RI) approximation.^56,57^ Empirical dispersion corrections were included with the DFT-D4 approach,^58^ as implemented in Turbomole.

This investigation concentrates on the E_2_ state of the FeMo cluster. Thus, we added two electrons and two protons to the resting E_0_ state, which is at the formal Mo^III^Fe_3_^II^Fe_4_^III^ oxidation state.^12,20,40,59^ It was studied in the quartet spin state, in agreement with experiments.^32−34^ Twenty-six different positions of the added protons were tested, as will be discussed below.

The electronic structure of all QM calculations was obtained with the broken-symmetry (BS) approach:^60^ Each of the seven Fe ions was modeled in the high-spin state, with either a surplus of α (four Fe ions) or β (three Fe ions) spin. Such a state can be selected in 35 different ways.^39^ The various BS states were obtained either by swapping the coordinates of the Fe ions^61^ or with the fragment approach by Szilagyi and Winslow.^62^ The various BS states are named by listing the number in the Noodleman nomenclature (BS1–10),^60^ followed by the numbers of the three Fe ions with minority spin.

### QM/MM Calculations

QM/MM calculations were performed with the ComQum software.^63,64^ In this approach, the protein and solvent are split into three subsystems: System 1 (the QM region) was relaxed by QM methods. System 2 contained all residues and water molecules with at least one atom within 6 Å of any atom in system 1 and it was optionally relaxed by MM. It included residues 59, 61, 62, 65–74, 92, 95–98, 191–199, 226–231, 234, 235, 253–255, 273–282, 300, 353–355, 358–364, 377–383, 385, 386, 401 422–427, 438, 440–444, 450, and 450 from subunit C and residues 97, 98, 101, and 105 from subunit D, in total 1488 atoms from 87 residues and 35 water molecules). Finally, system 3 contained the remaining part of the protein and the solvent, and it was kept fixed at the original coordinates (equilibrated crystal structure, to avoid the risk that different calculations end up in different local minima).

In the QM calculations, system 1 was represented by a wave function, whereas all the other atoms were represented by an array of partial point charges, one for each atom, taken from the MM setup. Thereby, the polarization of the QM system by the surroundings is included in a self-consistent manner (electrostatic embedding). When there is a bond between systems 1 and 2 (a junction), the hydrogen link-atom approach was employed: The QM system was capped with hydrogen atoms, the positions of which are linearly related to the corresponding carbon atoms (carbon link atoms, CL) in the full system.^63,65^ All atoms were included in the point-charge model, except the CL atoms.^66^ ComQum employs a subtractive scheme with van der Waals link-atom corrections.^67^ No cutoff is used for the QM and QM–MM interactions. However, for the optional MM optimization of system 2, a 30-Å cutoff for the nonbonded interactions had to be used. The geometry optimizations were continued until the energy change between two iterations was less than 2.6 J/mol (10^–6^ a.u.) and the maximum norm of the Cartesian gradients was below 10^–3^ a.u.

## Results and Discussion

### Relative Energies of Different Protonation States at the TPSS – BS7–235 Level

**Figure 2:**
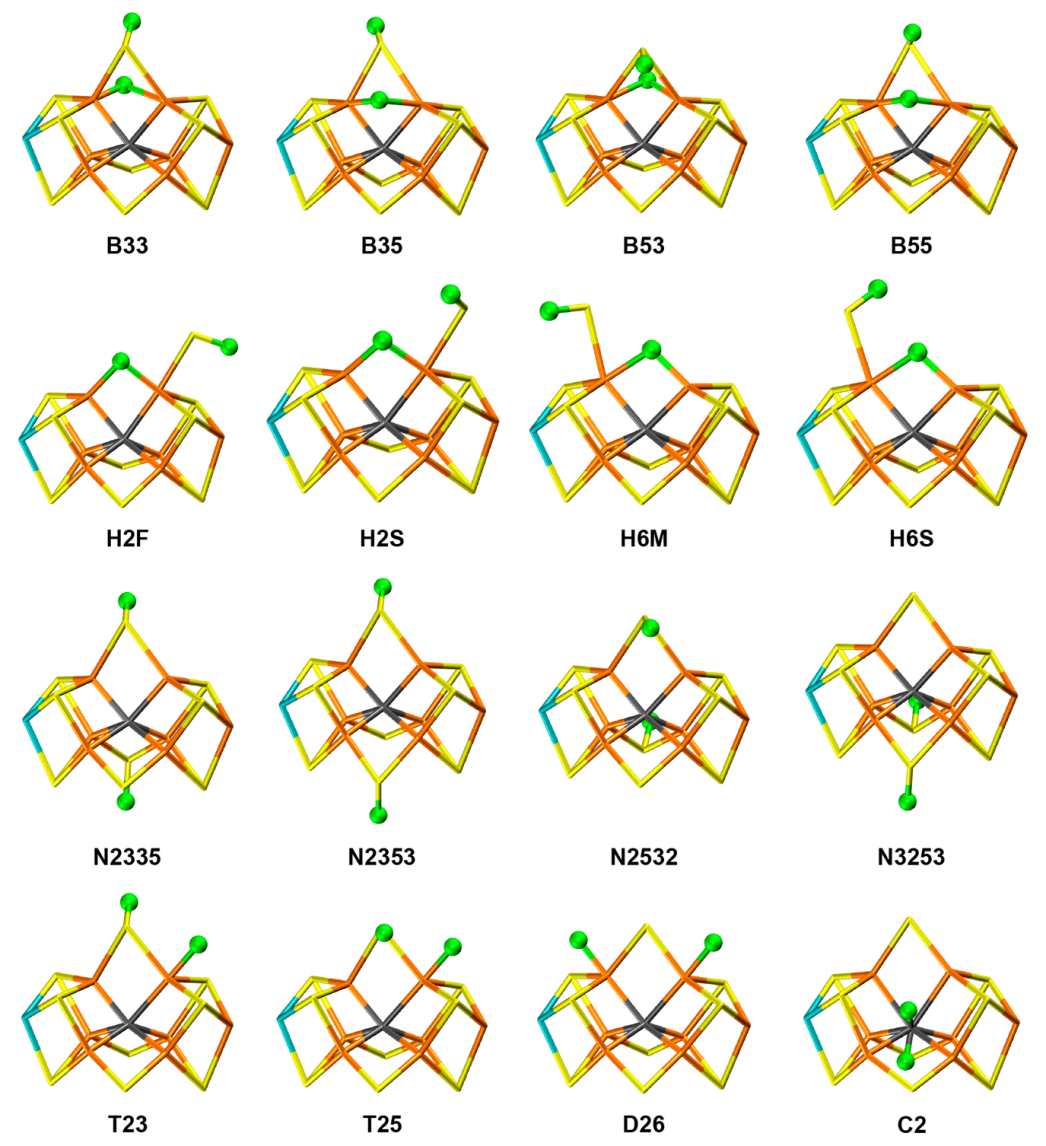
Sixteen of the 26 structures of the E_2_ state investigated in this work (all 26 structures are shown in Figure S2 in the Supporting Information).

In this investigation, we have examined 26 different possible structures for the E_2_ state, differing in the positions of the two added protons. We examined six types of structures (illustrated in Figure 2):

1. With a hydride ion bridging the Fe2 and Fe6 ions and the second proton on S2B, which is also bridging Fe2 and Fe6. The proton on S2B can point in two directions, viz. toward either S3A or S5A, called B3 and B5. Likewise, the hydride ion can be on either side of S2B (viz. on the same side as either S3A or S5A), giving the second number in our structure code, e.g., B35. The four conformations are shown in Figure 2. T&B called this type of structures bH(2,6)-CBS(S2B) and considered only three of the four conformations (not B53).
2. With a hydride ion bridging the Fe2 and Fe6 ions and a protonated S2B that is binding only to either Fe2 or Fe6. Thus, S2B is half-dissociated and the structures are called H2 and H6, depending on which Fe ion S2B still binds to. A second letter indicates whether the proton points toward Fe1, Mo, or a sulfide ion (F, M, or S; e.g., H2F). The four structures are shown in Figure 2. T&B called these structures bH(2,6)-OBS(2) or bH(2,6)-OBS(6) for H2S and H6S and considered only three of the four conformations (not H2S).
3. With two terminal hydride ions, one on Fe2 and the other on Fe6 (called D26 and shown in Figure 2). The hydride ions always bind trans to the central carbide ion.
4. With a terminal hydride ion on either Fe2 (T2) or Fe6 (T6) and a protonated and bridging S2B. Again, the proton on S2B can point in two directions, viz. toward either S3A or S5A, giving the second number in our structure code, e.g., T23, cf. Figure 2.
5. With no hydride ions, but instead the two protons on either S2B, S3A, or S5A. Each proton can point in two directions, viz. toward the other two of these three μ_2_ sulfide ions. The structures are named with a N (no hydride) followed by two pairs of numbers, the first indicating which sulfide is protonated and the second indicating the direction of that proton, e.g., N2532, indicating that S2B has a proton pointing toward S5A and S3A has the other proton pointing toward S2B (four examples are shown in Figure 2). T&B called these structures noH–CBS(S2B,S3B) and similar. They considered only four out of the 12 possible combinations and conformations.
6. With the two protons on the central carbide ion (C2). We considered only the state with the two protons directed toward the Fe2–Fe3–Fe6–Fe7 and Fe3–Fe4–Fe5–Fe7 faces of the cluster (shown in Figure 2), which was most favorable in our previous study.^27^

We have not studied states with S2B fully dissociated, because it is hard to accurately compare the energies of states with and without S2B, owing to uncertainties in the protonation state of S2B and the FeMo cluster, as well as the location of S2B after dissociation.

We first studied the 26 structures with TPSS-D4 and the BS-235 state. The results are shown in Table 1. It can be seen that the most stable structure is B33, i.e., with a bridging hydride between Fe2 and Fe6 on the side facing S3A and with the other proton on S2B (remaining bound to both Fe2 and Fe6), also pointing toward S3A. The structure with the S2B proton pointing in the opposite direction (B53) is only 4 kJ/mol less stable. Structures with the hydride bridge on the other side of S2B are 12 kJ/mol less stable than B33 if the S2B proton points toward S3B (B35), whereas if the S2B proton points in the opposite direction (B55), the structure is 33 kJ/mol less stable.

**Table 1:** Relative Energies (kJ/mol) of the 26 Structures, Calculated with TPSS and the BS7-235 State^a^

Structure	TPSS	TPSSh^38^
B33	0.0	19.4
B35	12.2	20.1
B53	4.2	
B55	32.9	37.5
H2F	75.1	35.0
H2S	83.9	
H6M	15.2	0.3
H6S	13.2	1.4
D26	13.3	37.3
T23	17.4	25.0
T25	46.2	33.0
T63	25.7	28.2
T65	37.2	35.3
N2352	35.5	
N2353	25.1	0.0
N2552	46.2	
N2553	33.6	14.1
N2332	58.1	
N2335	53.5	
N2532	72.7	68.7
N2535	63.7	
N3252	79.6	
N3253	72.6	48.4
N3552	76.7	
N3553	70.5	
C2	138.8	

a The third column shows the corresponding results from the T&B article (relative QM/MM energies with His-195 protonated on NE2 from Table S2), obtained with TPSSh.^38^.

We tested four structures with a half-dissociated protonated S2B ligand. The two structures with S2B dissociated from Fe6 are strongly unfavorable, 75–84 kJ/mol less stable than the B33 structure. However, the two structures with S2B dissociated from Fe2 are more stable, only 13–15 kJ/mol less stable than our best structure.

The structure with two terminal hydride ions on Fe2 and Fe6 (D26), as well as one structure with a terminal hydride ion on Fe2 (T23) are also competitive, 13 and 17 kJ/mol less stable than B33, respectively. However, the other structures with a terminal hydride on Fe2 or Fe6 are less stable, 26–46 kJ/mol worse than B33.

The 12 structures with both protons on the μ_2_ bridging sulfide ions (S2B, S5A, or S3A) are unfavorable. The best is N2353 (with S2B and S5A protonated, both protons pointing toward S3A), 25 kJ/mol less stable than B33. Structures with S2B and S3A protonated are worse, and those with S3A and S5A are worst, 71–80 kJ/mol less stable than B33, indicating that the preference of protonation follow the order S2B > S5A > S3A.

Finally, we also tested the C2 structure with a doubly protonated carbide ion. However, with TPSS it is strongly unfavorable, 139 kJ/mol higher in energy than B33.

These results are quite similar to what was found in our previous study,^27^ e.g., that the structure with a Fe2/6 bridging hydride and S2B protonated are most stable, better than structures with a terminal hydride or two protonated sulfide groups. However, the relative energies differ by up to 12 kJ/mol, owing to differences in the QM model and the BS state.

Table 1 includes also the results from T&B.^38^ It can be seen that the results diverge quite strongly from ours (by up to 40 kJ/mol). They reported three structures that are lowest in energy and nearly degenerate (within 1.4 kJ/mol), viz. one structure with S2B and S5A protonated (in our nomenclature N2353) and two structures with a bridging hydride ion and a protonated half-dissociated S2B (H6M and H6S). In our calculations these structures are 13–25 kJ/mol less stable than the best B33 structure. Likewise, T&B reported that the structures with both the hydride ion and S2B bridging Fe2 and Fe6 (they studied B33, B35, and B55) are 19–37 kJ/mol less stable than the N2354 structure. Naturally, such qualitative differences are alarming, considering that both studies use similar QM/MM methods. In the following sections, we examine possible reasons for this discrepancy.

### Effect of the BS States

T&B tested four different BS states for their structures, viz. the three BS7 states (BS7–235, BS7–247, and BS7–346) and the BS10–147 state.^38^ Still, energies for all four states are reported only for five structures, whereas only one state is reported for ten of the 24 structures studied. We decided to do a full BS investigation of all 35 BS states for one structure of each of the six types of structures mentioned above (and also for one of each of the three combinations of protonation of either S2B, S3A, and S5A, as well as for all four structures with half-dissociated S2B). For the other three structures of the same type, we investigated at least the BS7–235, BS7–247, BS7–346, and BS10–147 states and possibly additional states that were found to be low in energy for similar structures in the full investigation. In total 437 BS states were obtained with the TPSS-D4 functional for the 26 structures. The relative energies and Mulliken spin populations of the metal ions are listed in Table S1.

Table 2 shows the relative energies of the best BS state for the 26 different structures (second column). It can be seen that in most cases the change in relative energies is quite small, up 14 kJ/mol (20 kJ/mol for C2). Table 2 also shows the optimum BS state (third column). For 20 of the structures, it is one of the three BS7 states (but BS7–235 in only five cases). BS6–157 is most stable for the two half-dissociated H2F and H2S structures, whereas the BS2–234, BS8–347, BS10–127, and BS10–147 states are most stable for one structure each (D26, T65, N2532, and C2, respectively). In general, there are several BS states within 7 kJ/mol of the best one.

**Table 2:** Relative Energies (kJ/mol) of the 26 Different Structures, Calculated with Different Methods^a^

	TPSS						B3LYP	TPSSh		r^2^SCAN	
Structure	SV		TZ	r.e.	T&B	Relax	SV	SV		SV	
B33	5.1	235	5.3	**0.0**	11.7	25.4	119.7	10.8	157	31.7	247
B35	**0.0**	247	1.2	5.0	**0.0**	**0.0**	117.8	6.8	147	16.4	235
B53	8.4	346	**0.0**	8.5	17.4	4.8	136.1	24.2	346	34.3	247
B55	22.6	247	20.2	29.0	23.2	24.1	138.0	26.7	147	39.5	235
H2F	74.4	157	72.2	71.9	80.8	56.4	196.9	71.2	235	78.6	346
H2S	83.5	157	78.8	82.8	83.8	66.6	198.7	78.4	135	82.3	346
H6M	20.3	235	23.8	14.5	20.3	44.0	82.8	2.7	235	1.3	235
H6S	18.3	235	20.2	12.7	19.3	43.4	80.7	**0.0**	235	**0.0**	235
D26	15.3	234	10.6	6.6	16.1	38.6	181.4	45.3	235	44.5	235
T23	22.5	235	11.3	13.9	23.9	24.9	123.8	31.3	346	42.9	157
T25	39.8	247	27.3	37.7	40.8	32.6	138.1	41.7	346	50.6	157
T63	29.1	247	38.2	25.6	34.7	33.9	130.6	30.2	235	48.4	235
T65	41.9	347	35.9	39.1	45.6	35.7	134.4	41.0	235	58.1	235
N2352	29.9	247	16.6	30.3	39.0	31.6	99.8	21.5	247	52.7	247
N2353	18.2	346	26.1	16.8	30.0	19.3	86.6	14.2	346	46.6	147
N2552	48.0	247	33.7	51.6	63.2	42.8	113.6	38.8	346	59.2	346
N2553	29.7	346	37.6	31.8	40.6	44.3	95.8	22.1	247	49.0	346
N2332	57.9	247	36.4	66.1	66.7	61.8	115.9	50.0	247	68.7	235
N2335	49.8	346	43.5	58.0	62.1	86.6	74.0	27.8	235	60.0	147
N2532	71.8	127	121.2	79.2	114.0	97.8	124.3	45.0	235	76.2	235
N2535	61.6	346	56.8	72.5	71.4	63.5	104.0	47.4	346	69.1	346
N3252	84.6	235	65.4	86.3	98.8	90.6	150.9	82.3	346	96.1	346
N3253	75.1	346	63.4	81.6	84.6	82.3	129.3	66.5	247	84.7	346
N3552	75.8	346	60.2	84.6	86.5	80.9	132.1	64.9	247	86.8	346
N3553	61.7	346	47.6	71.2	73.0	65.0	122.6	56.1	346	75.6	346
C2	143.8	147	153.6	155.7	164.3	146.4	**0.0 (-27.2)^b^**	27.6	147	107.9	147
							b				

a Four different DFT methods were used, TPSS, B3LYP, TPSSh, and r^2^SCAN. All results were obtained with the def2-SV(P) basis set (SV), except those in the TZ column, which used the def2-TZVPD basis sets. In the r.e. column, relativistic effects were included. The “T&B” column shows the results with the smaller QM system used by T&B (still with TPSS). The “Relax” column shows the results obtained with the surrounding protein and water (within 6 Å of the QM system) allowed to relax by MM (also with TPSS). For TPSS, TPSSh, and r^2^SCAN, an investigation of the best BS state was performed and the best BS state is given after the energy, described by the three Fe ions with minority spin. The TZ, r.e., T&B, Relax, and B3LYP results were obtained for the same BS state as for TPSS. The most stable state for each method is marked in bold face.

b For BS8–345.

Most importantly, it can be seen from Table 2 that the BS-state investigation does not solve the discrepancy between our results and those of T&B: The B-type structures with both the hydride ion and the protonated S2B bridging Fe2 and Fe6 are lowest in energy, with B35 best, 5, 8, and 23 kJ/mol below B33, B53, and B55. D26 (with two terminal hydrides) is 15 kJ/mol less stable than B35. The two structures with S2B dissociated from Fe2 (H6M and H6S) are 18–20 kJ/mol higher in energy than B35, whereas the two structures with S2B dissociated from Fe6 are appreciably less stable (74–84 kJ/mol). The best structure with one terminal hydride ion (T23) is 23 kJ/mol less favorable than B35, and the best structure with no hydride ion (N2353) is 18 kJ/mol less favorable. The C2 structure is strongly disfavored. Thus, the BS states cannot explain the difference between our and the T&B results.

Table S1 also shows the TPSS spin populations on the metals. It can be seen that the largest Fe spin (in absolute terms) is 2.7–3.5 *e* (3.2 *e* on average). The average sorted spin populations decrease by ∼0.2 for the following four Fe ions, 2.9, 2.7, 2.6, and 2.4 *e*, still representing high-spin Fe. However, one or two of the Fe ions often have an appreciably lower spin population, as frequently observed for the FeMo cluster when hydride ions or other ligands coordinate to Fe:^18,27,44,68^ 75% of the studied structures and BS states have one Fe ion with a spin population below 2 *e* and 37% have two such Fe ions (2% have three).

For the best BS state of each structure, Fe1 always has the largest spin population. The half-dissociated structures and C2 have no Fe ion with a low spin (the lowest one is 2.2–2.8 *e*). For the other structures, Fe6 has a low spin population (0.2–1.8 *e*), except when S3A and S5A are both protonated (then instead Fe7 has a low spin population of 0.7–1.5 *e*). Sometimes, Fe7 (especially when S2B and S5A protonated), Fe5 (0.2 *e* for three structures with S2B and S3A protonated), or Fe2 (1.5–1.6 *e* for B53, T23, and T25) also have low spin populations. The spin population on Mo is small and negative, −0.2 *e* on average for the best BS states.

### Effect of the Basis Sets, Relativistic Effect, and the Model Size

Next, we studied how the basis sets affect the results. We calculated single-point energies for all 26 structures with the much larger def2-TZVPD basis set. The results in the fourth (TZ) column in Table 2 show that the basis set has a relatively small effect on the relative energies (as has also been observed before^27,69^); they change by up to 22 kJ/mol (−3 kJ/mol on average). The effect is largest for the complexes with two protonated sulfide ions, whereas those with a bridging sulfide ion change by less than 8 kJ/mol. The B53, B35, and B33 structures are still most stable and nearly degenerate (with 5 kJ/mol). However, the D26 and T23 structures are only 11 kJ/mol less stable, whereas the best half-dissociated structures (H6M and H6S) are 20–24 kJ/mol higher in energy.

Likewise, relativistic effects (estimated from the mass–velocity and Darwin terms) change the results by up to 12 kJ/mol (2 kJ/mol on average). The same three states (B33, B35, and B53) are still most stable, within 9 kJ/mol, but D26 is also within this range and the H6M and H6S half-dissociated structures are only 13–14 kJ/mol worse.

T&B used a smaller QM model than the one used in our study (144 atoms, compared to 191 atoms; cf. Figures 1 and S1). In particular, their model is missing the backbone of residues 355–359, which forms four hydrogen bonds to S3A, and two water molecules, which both form hydrogen bonds to S5A. To investigate the effect of this smaller QM model, we optimized the 26 protonation states also with their 144-atom model (still with the TPSS-D4 method and with the best BS state for the larger QM model). This had a rather small effect on the relative energies of the structures, 1–20 kJ/mol. In particular, all structures with no hydride ions are destabilized by 9–15 kJ/mol kJ/mol. However, the ordering of the relative stability of the structures is not changed. B35 is most stable, 12–19 kJ/mol more stable than B33, D26, B53, and H6S. Thus, the size of the QM model cannot explain the discrepancy between our and the T&B results.

### Relaxation of the Surrounding Protein

T&B allowed 1001 atoms surroundings the FeMo cluster to relax during the geometry optimization, whereas we by default keep all atoms outside the QM system fixed at the crystal structure (to avoid different states converge to different local minima of the surroundings). Even if most groups close to the S2B ligand are included in the QM system, it is possible that the surroundings may relax if S2B dissociates from one of the Fe ions, favoring such half-dissociated structures, especially as EPR experiments have indicated that some structural relaxation of the surroundings are involved in the conversion of the two observed E_2_ states.^34^ Therefore, we also run one set of calculations in which all residues with any atom within 6 Å of the QM system are relaxed by a MM optimization in every QM/MM geometry optimization step (1488 atoms). The results of these calculations are also included in Table 2 (column Relax).

It can be seen that this had a slightly larger effect on the relative energies, up to 37 kJ/mol. The two structures with S2B dissociated from Fe6 (H2F and H2S) are favored by 17–18 kJ/mol, but the two structures with S2B dissociated from Fe2 (H6M and H6S), as well as the D26 structure (with two hydride ion) are disfavored by 23–25 kJ/mol. Consequently, B35 is still the best structure, 5 kJ/mol better than B53, whereas N2353 is the third-best structure, 19 kJ/mol less stable than B35. The best half-dissociated structure, H6S, is 44 kJ/mol less stable than B35.

The movements of the surroundings are modest, with root-mean-squared movements of less 0.4 Å for the protein residues and somewhat larger for some water molecules (up to 0.7 Å). The movements are similar for all structures.

### Effect of the DFT Method

Previous studies have shown alarming differences between relative energies of nitrogenase FeMo cluster isomers calculated with different DFT methods.^23,27^ T&B also studied a few of their structures with both the TPSSh and the TPSS functionals^38^ and found quite large differences for the relative energies calculated with the two methods (up to 59 kJ/mol). Therefore, we reoptimized all our 26 structures also with the r^2^SCAN, TPSSh, and B3LYP functionals. The first is a meta-GGA functional that has been recommended for nitrogenase models,^70^ whereas the other two are hybrid functionals with 10 and 20% Hartree–Fock exchange, respectively. T&B used mainly TPSSh in their study. The results are also included in Table 2.

It can be seen that the DFT functional indeed has a strong influence on what E_2_ structure is preferred. B3LYP strongly prefers the C2 state, as was also previously observed.^27^ B3LYP also disfavors all states with Fe-bound hydride ions.

TPSSh has a smaller effect on the relative energies, up to 30 kJ/mol (besides the C2 structure, which is stabilized by 116 kJ/mol). It also somewhat disfavors structures with hydride ions and favors the half-dissociated structures, especially those for which S2B has dissociated from Fe2. Consequently, H6S becomes the best structure, 3 kJ/mol more stable than H6M and 7 kJ/mol more stable than the nondissociated B35 structure. The best structure with two protonated sulfide ions is N2353, 14 kJ/mol less stable than H6M. C2 is strongly stabilized, but it is still 28 kJ/mol less stable than H6S.

These results are based on the best BS state according to a restricted scan of a few of the best BS for TPSS (at least the BS7–235, BS7–247, BS7–346, and BS10–147 states and typically a few more states; a full investigation was performed on H6S; relative energies and Mulliken metal spin populations are shown in Table S2). The best BS state agrees with that suggested by T&B for seven of the 15 overlapping structures and they involve either the BS7 states or BS10–147 for all except two structures (BS6–157 for B33 and BS10–135 for HS2).

The Mulliken spin populations calculated by TPSSh are in general larger and more even than those obtained with TPSS. The average absolute values of the sorted spin populations are 3.6, 3.5, 3.4, 3.3, 3.2, 2.9, and 2.6 *e*, i.e., 0.4–1.2 *e* larger than for the corresponding TPSS spin populations. No structure has two Fe ions with a low (<2 *e*) spin population, but 15% of the structures have one Fe ion with a spin population less than 2 *e*. However, among the best BS states, only one structure (B33) has a Fe spin population of 2.0 *e*; for the other structures, the lowest Fe population is 2.4–3.3 *e*. Thus, with TPSSh, a low spin population typically indicates convergence to a suboptimal wave function or BS. Our TPSSh spin populations are typically ∼0.2 *e* larger than those reported by T&B,^38^ reflecting differences in the QM model, the basis sets, and other details of the calculations. The difference is never larger than 0.5 *e*. The Mo spin population for the best BS state is −0.2 to −0.7 *e* (average −0.4 *e*) with a single exception (B33 has a positive population of 0.5 *e*).

The results obtained with the r^2^SCAN functional are similar. Compared to TPSSh, the half-dissociate structures, as well as D26 (with two terminal hydrides), are stabilized relative to the other states. Consequently, the two half-dissociated H6S and H6M structures (which are essentially degenerate) are most stable, followed by B35, which is 16 kJ/mol less stable. The best structures with terminal hydrides (T23 and D26) are 43–44 kJ/mol less stable, and the best structure with two protonated sulfide ions (N2353) is 47 kJ/mol less stable than H6S.

As with TPSSh, we made a limited investigation of the most stable BS state with r^2^SCAN (shown in Table S3). There are some variations compared to TPSS and TPSSh, but mainly within the BS7 and BS10–147 states. However, T23 and T25 are most stable in the BS6–157 state.

The Fe spin populations of r^2^SCAN are very similar to those of TPSSh with an average difference of only 0.04 *e*. However, the spin population on Mo for the best BS state varies much more than for the other two methods. It is positive (0.4–0.9 *e*) for three structures, and it is −1.4 to −1.6 *e* for seven of the structures with two protonated sulfide ions.

Considering that at the TPSS level, relaxation of the surroundings had a quite large effect on the relative energies and destabilized the best half-dissociated structures by ∼25 kJ/mol, we also ran QM/MM geometry optimizations with relaxed surroundings with the TPSSh and r^2^SCAN functionals for the eight best structures. The results in Table S4 show that in this case, relaxation of the surrounding has rather small effects, up to 10 kJ/mol, except that B35 is selectively destabilized by both methods (by 19–33 kJ/mol). Thus, the half-dissociated structures remain most favorable.

## Conclusions

In this study, we have tried to explain the discrepancy between the results obtained by our group^27^ and those obtained by T&B^38^ regarding the relative energies of the E_2_ state of nitrogenase and, in particular, the importance of half-dissociated states with the S2B ligand dissociated from either Fe2 or Fe6. Our results show that the BS state, the basis set, relativistic effects, the size of the QM model, and the relaxation of the surrounding have some influence on the relative stabilities (by up to 12, 22, 9, 20, and 37 kJ/mol, respectively). Our calculations also emphasize the importance of studying all conformations of the added protons, which may change the relative energies by up to 33 kJ/mol. However, neither of these variations changes the relative ordering of the different types of structures. Instead the difference between the two studies is caused by the use of different DFT methods:

- TPSS favors structures with both the hydride and S2B bridging Fe2 and Fe6 (B35, B33, and B53), which are 15–18 kJ/mol better than structures with a half-dissociated S2B (H6S), with two terminal hydride ions (D26) or with no hydride ions (N2353).
- B3LYP strongly favors the C2 structure with a doubly protonated carbide ion, which is 101 kJ/mol more stable than N2335. It strongly disfavors all structures with Fe-bound hydride ions.
- TPSSh also shows similar tendencies, but to a smaller extent (owing to the smaller amount of Hartree–Fock exchange). It also favors the half-dissociated structure so that H6S and H6M becomes 7 kJ/mol more stable than B35. This shows that the Hartree–Fock exchange weakens the Fe–S and Fe–H bonds.
- r^2^SCAN selectively favors the half-dissociated structures and C2 (but the latter much less than the hybrid functionals). Therefore, H6S and H6M become 16 kJ/mol more stable than B35.

**Figure 3:**
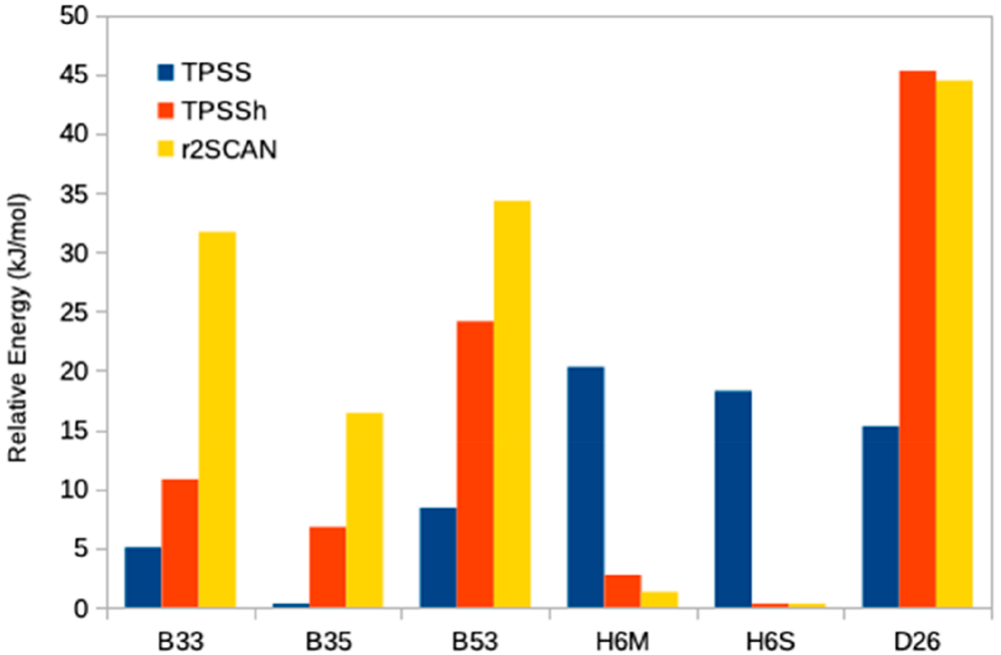
Relative stabilities of the best structures containing hydride ions (in agreement with experimental data^33,34^) for the TPSS, TPSSh, and r^2^SCAN methods.

From Table 2, it can be seen that eight E_2_ structures (B35, B33, B53, H6M, H6S, D26, N2353, and C2) are competitive in terms of energies (within 20 kJ/mol at least with some DFT method). However, two of them (C2 and N2353) do not contain iron-bound hydride ions and are therefore not compatible with the EPR data.^33,34^ This calls in doubt the B3LYP calculations, which strongly prefer the C2 structure. The relative energies of the remaining five structures obtained with TPSS, TPSSh, and r^2^SCAN are shown in Figure 3. It can be seen that the D26 structure is not preferred with any method and is relatively high in energy. The other structures all have a hydride ion bridging Fe2 and Fe6, showing that E_2_ most likely contains such a bridging hydride ion. The low-energy structures are of two types: either with S2B also bridging Fe2 and Fe6 (B33, B35, and B53) or with S2B dissociated from Fe2 (H6M and H6S). Within these groups, the structures differ only in the direction of the proton on S2B and (for B-type structures) which side of S2B the hydride ion is. All three methods agree that there are at least one more low-energy structure of the same type within 1–5 kJ/mol, in agreement with the observation of two nearly degenerate structures in the EPR experiments.^33,34^ Thus, the only disagreement is that TPSS points out the nondissociated S2B as the best, whereas TPSSh and r^2^SCAN prefer the half-dissociated structures, with energy difference of 7–18 kJ/mol toward the other structure.

A natural question is then which DFT functional to trust. Recent studies have indicated that r^2^SCAN, TPSSh, and B3LYP* (with 15% Hartree–Fock exchange) give the best structures of Fe_2_ and FeMo models.^70^ On the other hand, another study indicated that pure GGA functionals, like PBE and PW91, gave the best results for structures and energies involving the binding of H_2_ and N_2_ to small transition-metal models with relation to nitrogenase.^71^ Yet, further studies on the latter systems with dispersion-corrected DFT functionals indicated that pure GGA functionals give better structures, whereas hybrid functionals give more reliable energetic results.^72^ Consequently, further investigation are required to decide which DFT functional gives the most reliable results for nitrogenase models. However, considering the small energy difference between the two types of structures with most methods, it is clear that both types need to be considered in investigations of the mechanism of nitrogenase.

## Supporting Information Available

Figure of the 144-atom T&B QM model; figure of all 26 structures; relative energies and Mulliken spin populations for the various BS states for the 26 structures, calculated with TPSS, TPSSh, and r^2^SCAN (PDF)
Coordinates of all studied structures (ZIP)
