# Supplementary material for: QM/MM Study of Partial Dissociation of S2B for the E2 Intermediate of Nitrogenase
Source: Inorg Chem. 2022 Oct 28;61(45):18067–76. doi: 10.1021/acs.inorgchem.2c02488 (PMC9667496; doi:10.1021/acs.inorgchem.2c02488)
Supplement: Supplementary file 1 — ic2c02488_si_001.pdf [file ic2c02488_si_001.pdf]

Supporting Information

QM/MM study of partial dissociation of S2B for the E_2 intermediate of nitrogenase

Hao Jiang, Oskar K. G. Svensson and Ulf Ryde *

Department of Theoretical Chemistry, Lund University, Chemical Centre, P. O. Box 124, SE-221 00 Lund, Sweden

Correspondence to Ulf Ryde, E-mail: Ulf.Ryde@teokem.lu.se,

Tel: +46 – 46 2224502, Fax: +46 – 46 2228648

2022-10-06

Figure S1. Structure of the FeMo cluster (B33 state) with the 144-atom T&B model.

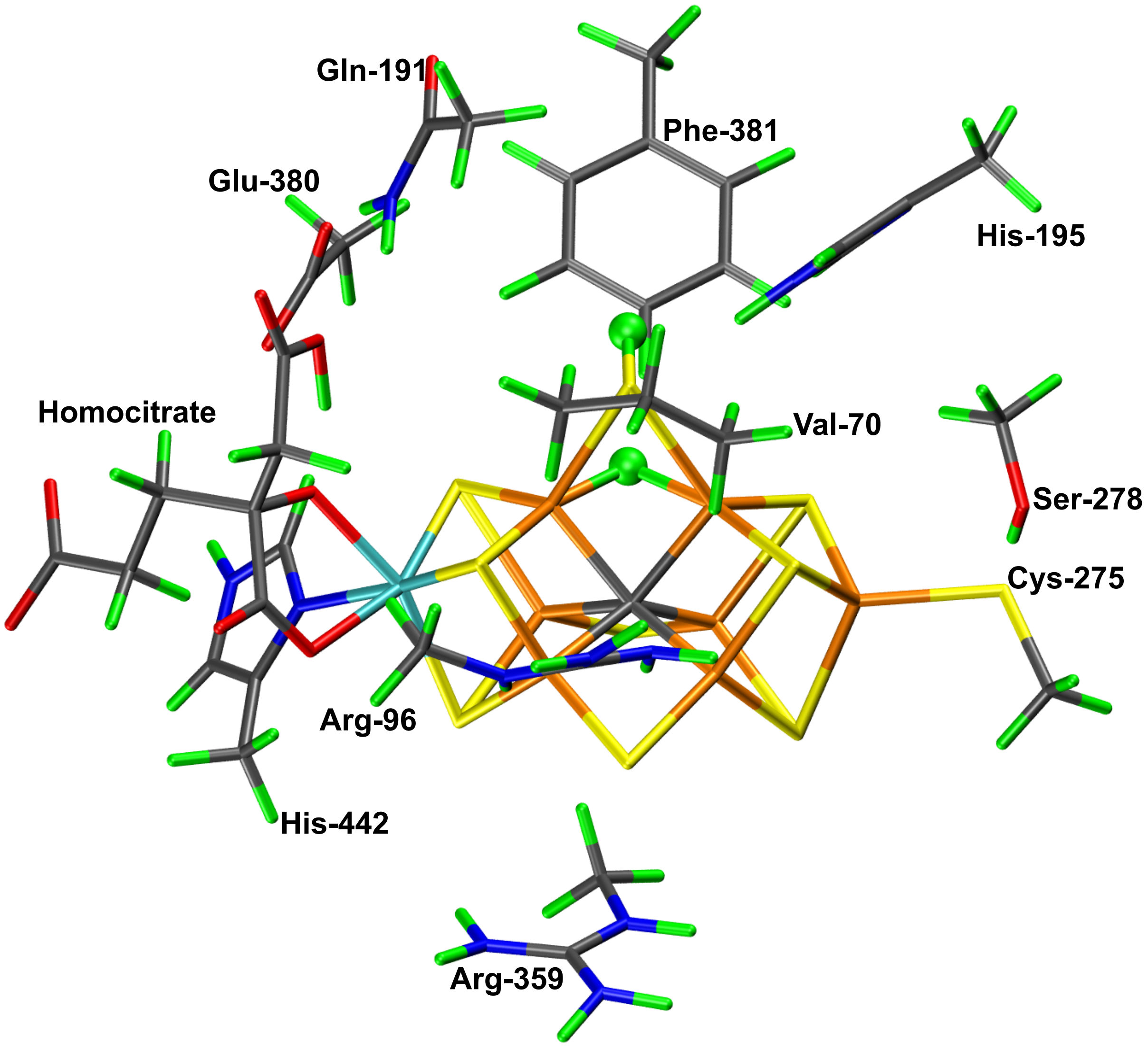

Figure S2. The 26 structures of the E_2 state investigated in this study.

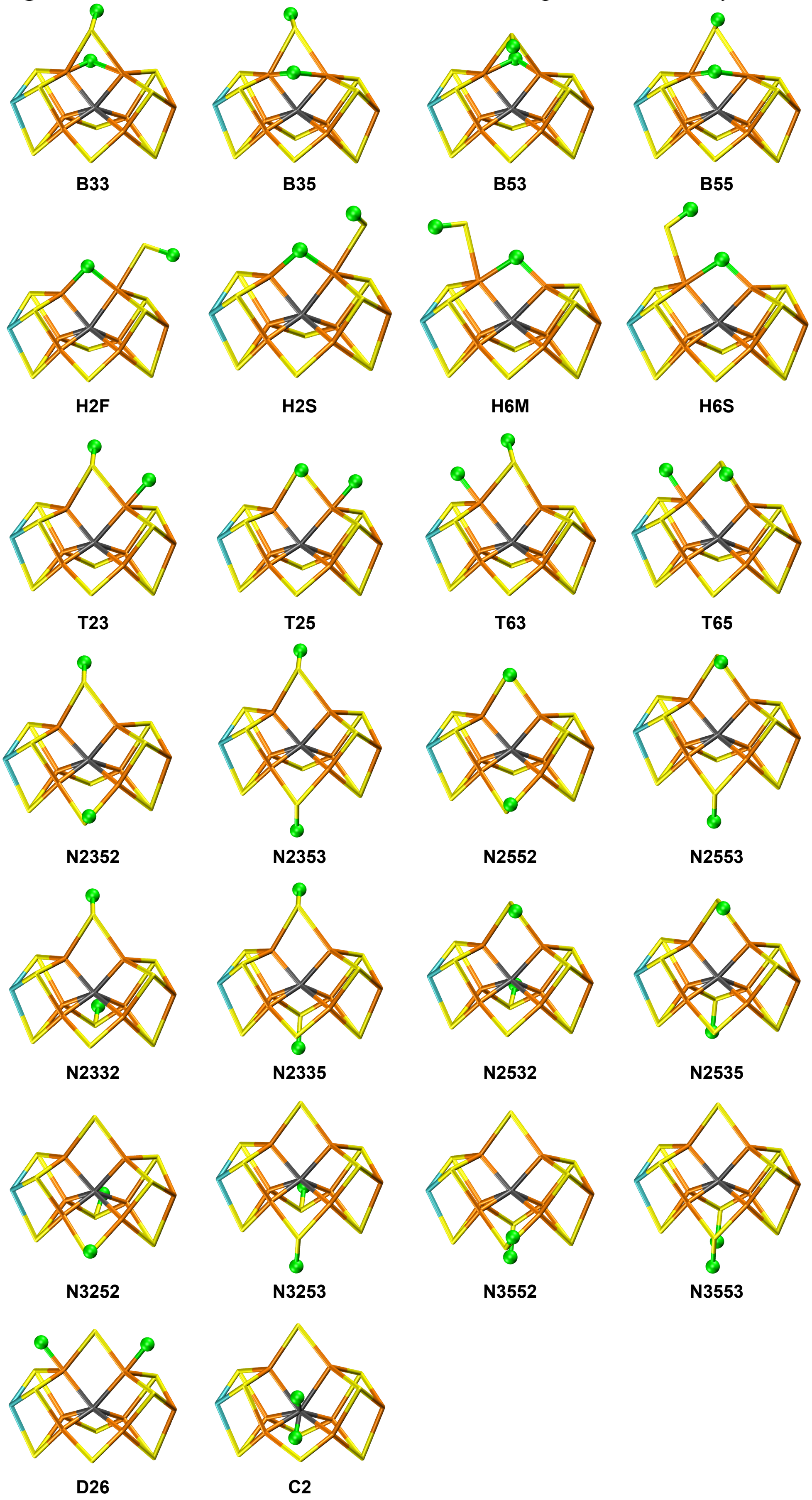

Table S1. Relative energies (kJ/mol) for the various BS states for the 26 structures, calculated with TPSS (ΔE_s within the same structure, ΔE for all structures and BS states). The Mulliken spin population for all Fe ions and Mo are also listed.

Structure	BS	ΔE_S	ΔE	Fe1	Fe2	Fe3	Fe4	Fe5	Fe6	Fe7	Mo
B33	257	37	42	3.3	-3.0	3.1	3.1	-2.3	0.1	-2.0	0.5
	467	24	29	3.3	1.2	2.9	-2.9	2.8	-2.2	-2.5	0.2
	456	57	62	3.3	1.7	2.8	-2.9	-2.5	-2.6	2.5	0.3
	457	44	49	3.3	-2.3	3.0	-2.9	2.7	1.6	-2.5	-0.2
	246	11	16	2.9	-3.0	0.4	-3.0	2.7	-1.1	2.1	-0.3
	247	11	16	3.2	-2.6	3.1	-2.8	2.8	0.9	-1.7	-0.3
	245	33	38	3.2	-2.6	3.1	-2.5	-2.3	1.2	2.7	-0.2
	236	16	21	3.3	-2.8	-3.1	2.8	1.6	-1.5	2.4	-0.1
	237	39	44	3.2	-2.7	-2.6	3.0	2.9	1.3	-1.9	-0.3
	235	0	5	3.3	-2.6	-2.8	3.1	-2.7	0.2	2.3	0.0
	346	5	10	3.3	0.8	-2.8	-2.8	2.6	-2.5	2.4	-0.2
	347	12	17	3.2	2.4	-2.1	-2.9	2.9	2.1	-2.3	-0.4
	345	4	9	3.3	2.9	-2.8	-2.3	-2.2	1.5	2.8	-0.3
	234	28	33	3.2	-1.2	-2.7	-2.8	2.6	1.9	2.4	-0.7
	234	48	53	3.2	-2.1	-2.5	-2.5	3.0	1.7	2.6	-0.8
	167	23	28	-3.3	2.2	2.7	2.8	2.1	-0.6	-2.8	0.2
	157	38	43	3.3	-1.8	-2.6	-2.7	2.8	-0.6	2.6	-0.5
	126	17	22	-3.3	-3.1	2.8	2.9	2.2	-0.5	2.1	-0.3
	127	20	25	-3.3	-0.7	2.8	3.1	2.2	2.1	-2.8	-0.2
	137	40	45	-3.2	2.7	-2.7	3.0	3.0	2.4	-1.0	-0.6
	135	40	45	-3.3	2.7	-3.0	3.0	-2.4	1.9	2.7	-0.3
	147	38	43	-3.3	2.7	3.0	-2.8	2.9	2.7	-1.5	-0.5
	145	41	46	-3.3	3.0	3.1	-2.3	-1.8	2.0	2.8	-0.4
	124	76	81	-3.2	-2.8	2.9	-0.3	3.0	1.3	2.8	-0.8
	134	61	66	3.4	-1.9	3.2	3.2	-2.3	-2.1	-2.0	0.7
	14	9	14	-3.2	2.6	2.5	-3.2	2.6	0.1	2.6	-0.6
	15	21	26	-3.3	1.9	2.0	2.6	-2.9	1.2	1.9	-0.1
B35	247	0	0	3.2	-2.6	3.1	-2.8	2.7	0.9	-1.9	-0.2
	235	17	17	3.3	-2.6	-2.8	3.1	-1.9	1.0	2.7	-0.3
	346	7	7	3.3	1.5	-2.8	-2.8	2.6	-1.3	2.5	-0.4
	234	40	40	3.2	-2.2	-2.2	-2.6	2.7	1.6	2.9	-0.8
	157	25	25	-3.1	3.0	2.9	3.1	-2.4	1.8	-2.4	0.2
	135	46	46	-3.2	2.7	-2.8	3.0	-1.7	2.6	2.7	-0.5
	147	36	36	-3.3	3.2	3.1	-2.7	2.9	2.1	-1.9	-0.4
B53	247	23	31	3.2	-1.6	3.0	-2.8	2.5	1.2	-2.6	-0.1
	235	1	9	3.2	-2.4	-2.8	3.1	-2.1	0.9	2.6	-0.2
	346	0	8	3.3	1.6	-2.7	-2.8	2.6	-1.4	2.5	-0.4
	157	26	34	-2.9	2.6	2.9	2.9	-2.6	2.3	-2.3	0.2
	135	42	51	-3.2	3.0	-2.5	3.1	-2.1	2.2	2.7	-0.3
	147	35	44	-3.2	2.6	3.0	-2.8	2.9	2.7	-1.5	-0.5

B55	247	0	23	3.2	-2.6	3.1	-2.8	2.7	0.9	-1.8	-0.2
	235	15	38	3.3	-2.6	-2.8	3.1	-1.9	1.0	2.7	-0.3
	346	4	26	3.3	1.7	-2.8	-2.8	2.7	-1.4	2.5	-0.4
	157	42	65	3.2	-2.2	-2.3	-2.6	2.8	1.6	2.9	-0.8
	135	38	61	-3.2	2.6	-2.8	3.0	-1.7	2.7	2.7	-0.5
	147	32	54	-3.3	3.1	3.1	-2.7	2.9	2.3	-1.9	-0.4
	34	29	52	3.1	0.6	-2.8	-2.8	2.6	0.1	2.4	-0.5
H2F	567	99	174	2.6	2.6	2.8	1.7	-2.8	-2.5	-2.4	0.9
	256	62	137	3.0	-2.5	1.5	2.8	-2.7	-2.6	2.5	0.3
	367	49	123	3.3	2.9	-2.7	1.7	2.5	-2.6	-2.5	0.4
	356	30	104	3.2	2.5	-2.9	2.4	-2.7	-2.5	2.6	0.3
	467	43	117	3.2	2.0	2.7	-2.8	2.6	-2.5	-2.6	0.3
	456	58	132	3.2	2.9	1.7	-2.6	-2.7	-2.6	2.5	0.3
	457	63	138	3.1	1.5	3.1	-2.6	-2.5	2.6	-2.5	0.1
	246	30	105	3.2	-2.5	2.7	-3.0	2.7	-2.5	2.4	-0.1
	247	9	83	3.2	-2.4	2.9	-2.8	2.6	2.3	-2.7	-0.2
	245	38	112	3.1	-2.4	2.7	-2.6	-2.6	2.5	2.4	-0.3
	236	23	98	3.3	-2.5	-3.0	2.5	2.6	-2.5	2.5	-0.1
	237	38	112	3.2	-2.6	-3.0	2.6	2.7	2.6	-2.3	-0.3
	235	6	80	3.2	-2.4	-2.8	2.8	-2.7	2.4	2.5	-0.2
	346	11	85	3.3	2.9	-2.8	-2.8	2.5	-2.6	2.3	-0.1
	345	28	102	3.2	2.8	-2.8	-2.0	-2.5	2.3	2.5	-0.3
	234	11	85	3.2	-1.9	-2.7	-2.6	2.6	2.4	2.6	-0.7
	156	7	82	-3.2	2.1	3.0	2.8	-2.5	-2.3	2.7	0.2
	167	5	79	-3.2	2.1	2.9	3.0	2.7	-2.3	-2.5	0.3
	157	0	74	-3.2	2.4	2.8	2.7	-2.3	2.8	-2.2	0.0
	126	39	114	-3.3	-2.4	3.0	3.0	2.5	-2.3	2.3	-0.2
	125	31	106	-3.3	-2.5	3.1	2.9	-2.5	2.7	2.6	-0.4
	136	53	128	-3.2	2.6	-3.0	2.9	2.9	-1.9	2.7	-0.4
	137	37	111	-3.1	2.3	-2.2	2.7	2.8	2.9	-2.0	-0.5
	135	20	94	-3.2	2.6	-2.8	2.9	-1.8	3.0	2.7	-0.5
	146	48	122	-3.2	2.6	3.1	-2.9	2.8	-2.0	2.7	-0.4
	147	12	86								
	145	31	105	-3.2	2.4	3.0	-2.0	-2.3	2.8	2.7	-0.5
	123	64	138	-2.7	-1.0	-2.6	2.6	2.7	2.6	2.2	-0.8
	134	68	143	-2.9	2.5	-1.7	-2.3	2.9	2.9	2.6	-0.9
H2S	567	80	163	2.4	2.4	1.9	2.8	-2.8	-2.2	-2.4	0.7
	256	58	142	2.9	-2.4	1.5	2.8	-2.7	-2.6	2.5	0.3
	356	29	113	3.2	2.5	-2.9	2.4	-2.7	-2.5	2.6	0.3
	467	37	120	3.2	2.2	2.7	-2.9	2.6	-2.6	-2.6	0.3
	456	50	134	3.2	2.9	1.6	-2.7	-2.7	-2.5	2.4	0.3
	457	71	155	3.2	1.1	3.2	-2.6	-2.3	2.5	-2.3	0.0
	246	23	107	3.2	-2.5	2.6	-3.0	2.7	-2.5	2.4	-0.2
	247	7	90	3.2	-2.5	2.9	-2.8	2.6	2.3	-2.6	-0.2
	245	35	118	3.2	-2.5	2.8	-2.7	-2.6	2.5	2.5	-0.3

	237	37	120	3.2	-2.7	-3.0	2.7	2.7	2.6	-2.4	-0.3
	235	5	89	3.2	-2.4	-2.8	2.9	-2.7	2.3	2.5	-0.2
	346	8	91	3.3	3.0	-2.8	-2.8	2.5	-2.6	2.3	-0.1
	234	11	94	3.2	-1.9	-2.7	-2.6	2.6	2.3	2.6	-0.7
	156	6	90	-3.2	2.2	3.0	2.8	-2.5	-2.2	2.6	0.2
	167	5	88	-3.2	2.1	2.9	3.0	2.7	-2.3	-2.5	0.2
	157	0	84	-3.2	2.4	2.8	2.7	-2.3	2.8	-2.2	0.0
	126	34	118	-3.3	-2.5	3.0	3.0	2.5	-2.2	2.3	-0.2
	127	31	114	-3.3	-2.4	2.9	3.0	2.6	2.6	-2.3	-0.3
	125	51	134	3.2	3.2	-1.9	-1.8	2.9	-1.1	-2.0	0.0
	136	47	130	-3.2	2.8	-3.0	2.9	2.8	-1.7	2.7	-0.4
	137	38	121	-3.1	2.4	-2.5	2.8	2.7	2.9	-1.8	-0.4
	135	17	100								
	146	60	144	3.2	-1.3	-2.4	3.2	-2.4	2.8	-0.2	-0.1
	147	9	92	-3.3	2.6	3.0	-2.8	2.8	2.9	-1.7	-0.5
	145	73	157	3.2	-1.8	-1.5	3.1	2.8	-1.8	-1.8	0.1
	123	86	174	3.5	2.9	3.2	-1.6	-2.4	-2.0	-2.1	0.6
	134	59	147	-2.9	2.5	-1.7	-2.3	2.9	2.9	2.6	-0.9
H6M	567	109	129	1.7	2.5	2.4	3.1	-2.6	-2.3	-2.7	0.8
	256	55	75	3.0	-2.8	2.2	3.0	-2.8	-2.6	2.2	0.4
	257	45	65	2.8	-2.8	2.0	2.9	-2.7	2.8	-2.4	0.2
	367	66	86	3.2	2.8	-2.9	2.6	2.2	-2.7	-2.6	0.4
	467	43	63	2.9	2.0	3.0	-3.0	2.7	-2.4	-2.5	0.3
	456	65	86	2.7	2.9	2.6	-2.8	-2.3	-2.6	2.3	0.3
	457	61	81	3.3	2.4	3.1	-3.0	-2.8	2.3	-2.6	0.3
	246	30	50	3.2	-2.6	2.9	-3.0	2.7	-2.5	2.0	-0.2
	247	2	23	3.2	-2.7	3.0	-2.9	2.5	2.4	-2.6	-0.3
	245	24	45	3.2	-2.7	3.0	-2.9	-2.4	2.6	2.2	-0.3
	236	33	54	3.2	-2.6	-3.0	2.9	2.3	-2.6	2.6	-0.1
	237	26	46	3.2	-2.8	-3.0	3.0	2.3	2.6	-2.3	-0.3
	235	0	20	3.3	-2.7	-2.8	3.0	-2.7	2.4	2.4	-0.2
	346	13	34	3.2	3.0	-2.7	-2.8	2.6	-2.8	2.5	-0.2
	347	-10	10	3.3	2.9	-2.3	-2.9	2.9	1.6	-2.2	-0.3
	345	25	45	3.3	3.1	-2.9	-2.9	-2.4	2.1	2.7	-0.2
	234	18	38	3.2	-2.6	-2.7	-2.7	2.8	2.5	2.8	-0.9
	156	14	35	-3.1	2.8	2.9	2.9	-2.6	-2.4	2.2	0.4
	167	17	37	-3.2	2.7	2.7	3.0	2.4	-2.3	-2.5	0.3
	157	12	32	-3.2	2.5	3.0	3.0	-2.6	2.7	-2.4	0.2
	126	38	58	-3.2	-2.9	2.6	2.7	2.9	-1.5	2.8	-0.5
	127	43	64	-3.2	-2.9	3.0	3.1	2.9	2.7	-2.1	-0.5
	125	47	67	-3.3	-2.9	3.1	3.0	-2.4	2.7	2.8	-0.4
	136	36	57	-3.2	2.6	-3.1	3.0	2.8	-1.1	2.4	-0.4
	137	37	57	-3.2	2.8	-2.8	3.1	2.8	2.4	-1.6	-0.4
	135	41	62	-3.2	3.0	-2.4	3.0	-2.4	2.4	2.7	-0.3
	146	63	83	3.1	-0.9	-2.4	3.2	-2.1	3.1	-1.4	0.0

	147	38	58	-3.2	3.0	3.0	-2.6	2.8	2.5	-2.1	-0.5
	145	42	62	-3.2	2.9	3.1	-2.7	-1.9	2.4	2.7	-0.4
	124	67	87	-2.9	-1.7	3.0	-3.0	2.9	2.5	2.7	-0.9
	123	72	93	-2.9	-1.3	-2.9	2.9	2.8	2.4	2.6	-0.9
	134	65	86	-3.0	2.8	-1.2	-2.8	2.8	2.5	2.6	-0.9
H6S	567	110	129	1.7	2.6	2.5	3.1	-2.6	-2.3	-2.7	0.8
	267	61	79	3.1	-2.8	2.9	2.2	2.3	-2.6	-2.7	0.3
	256	52	70	3.1	-2.8	2.2	3.0	-2.8	-2.6	2.3	0.4
	257	48	66	2.8	-2.8	2.1	2.9	-2.7	2.8	-2.4	0.2
	367	67	86	3.2	2.8	-2.9	2.5	2.3	-2.7	-2.7	0.4
	356	39	57	3.0	2.0	-2.9	3.0	-2.6	-2.4	2.6	0.3
	467	44	62	2.9	2.1	3.0	-3.0	2.7	-2.4	-2.5	0.3
	456	67	85	2.7	2.9	2.6	-2.8	-2.3	-2.6	2.3	0.3
	457	30	49	3.4	2.0	3.1	-2.8	-2.5	2.3	-2.7	0.2
	246	33	51	3.2	-2.6	2.9	-3.0	2.7	-2.5	2.0	-0.2
	247	4	22	3.2	-2.7	3.0	-2.9	2.5	2.4	-2.6	-0.3
	245	26	45	3.2	-2.7	3.0	-2.9	-2.4	2.6	2.2	-0.3
	236	37	56	3.3	-2.6	-3.0	2.9	2.3	-2.6	2.5	-0.2
	237	27	46	3.3	-2.9	-3.0	3.0	2.3	2.5	-2.3	-0.3
	235	0	18	3.3	-2.7	-2.9	3.0	-2.7	2.5	2.5	-0.2
	346	18	36	3.2	3.0	-2.7	-2.8	2.7	-2.9	2.4	-0.2
	347	27	46	3.3	3.0	-2.9	-3.0	2.7	2.4	-2.3	-0.3
	345	27	46	3.3	3.1	-2.9	-2.9	-2.4	2.3	2.7	-0.2
	234	19	37	3.2	-2.6	-2.7	-2.7	2.8	2.5	2.8	-0.9
	156	15	33	-3.1	2.8	2.9	2.9	-2.6	-2.4	2.2	0.4
	167	18	36	-3.2	2.8	2.8	3.0	2.4	-2.3	-2.5	0.3
	157	17	35	-3.2	2.6	3.0	3.0	-2.6	2.7	-2.4	0.2
	126	50	68	-3.2	-2.8	2.8	2.9	2.9	-2.1	2.6	-0.4
	127	46	65	-3.2	-2.9	3.0	3.1	2.9	2.7	-2.2	-0.5
	125	47	66	-3.3	-2.9	3.1	3.0	-2.5	2.7	2.8	-0.4
	136	41	59	-3.2	2.7	-3.0	3.0	2.9	-1.5	2.5	-0.4
	137	39	57	-3.2	2.8	-2.8	3.1	2.8	2.4	-1.7	-0.4
	135	40	58	-3.2	3.0	-2.5	3.0	-2.5	2.6	2.7	-0.3
	146	41	59	-3.2	2.8	3.0	-3.0	2.7	-2.0	2.8	-0.3
	147	38	56	-3.2	3.0	3.0	-2.6	2.8	2.5	-2.1	-0.4
	145	42	60	-3.2	2.9	3.1	-2.8	-2.0	2.5	2.7	-0.3
	124	67	86	-2.9	-1.7	3.0	-3.0	2.8	2.6	2.7	-0.8
	123	77	96	-2.8	-1.9	-2.7	3.0	2.9	2.3	2.8	-0.8
	134	64	82	-3.0	2.9	-1.2	-2.8	2.8	2.5	2.7	-0.9
D26	357	56	71	3.0	1.3	-2.6	3.1	-2.6	1.7	-1.3	0.2
	247	5	20	3.2	-2.3	2.8	-2.8	2.6	1.9	-2.4	-0.2
	237	34	49	3.2	-2.4	-2.8	2.8	2.5	1.9	-2.2	-0.2
	235	3	18	3.2	-2.3	-2.8	2.9	-2.5	1.9	2.5	-0.2
	346	26	41	3.3	2.5	-2.6	-2.7	2.5	-2.1	2.2	-0.2
	347	63	78	3.1	0.4	-1.8	-2.5	2.9	2.0	-1.0	-0.4

	234	0	15	3.2	-2.1	-2.5	-2.5	2.8	1.8	2.6	-0.7
	156	1	16	-3.1	2.2	2.9	2.7	-2.5	-1.7	2.3	0.2
	167	3	19	-3.2	2.2	2.6	2.9	2.4	-1.6	-2.5	0.2
	126	32	48	-3.2	-2.5	2.4	2.4	2.7	-1.2	2.6	-0.5
	127	41	56	-3.2	-1.7	2.8	3.0	2.5	1.9	-2.4	-0.2
	125	62	77	3.1	2.6	-1.7	-1.9	3.0	-0.5	-1.8	-0.1
	136	32	47	-3.2	2.3	-2.8	2.6	2.8	-0.7	2.4	-0.6
	137	32	48	-3.2	2.4	-2.3	3.0	2.9	2.0	-1.7	-0.3
	135	44	60	3.2	-1.4	3.1	-2.4	2.7	-0.7	-1.8	-0.1
	146	63	78	3.2	-0.8	-2.2	3.2	-2.1	2.2	-0.8	0.1
	147	40	55	-2.9	2.3	2.7	-1.0	2.6	1.9	-2.5	-0.2
	145	36	51	-3.2	2.4	3.1	-2.3	-1.8	2.1	2.8	-0.3
	123	108	123	3.5	2.7	3.3	-1.4	-2.5	-1.8	-1.9	0.6
	134	79	94	3.3	-2.3	3.1	3.2	-1.8	-1.8	-1.7	0.5
T23	257	40	63	3.2	-2.1	2.7	2.9	-2.7	1.1	-2.6	0.5
	357	36	59	3.1	0.8	-2.5	3.2	-2.5	2.0	-1.3	0.0
	467	34	56	3.3	2.5	-2.9	2.9	-2.8	2.3	-2.4	0.3
	457	53	76	3.4	2.6	3.2	-2.8	-2.8	1.3	-2.5	0.3
	247	3	26	3.2	-1.6	3.2	-2.6	2.6	0.3	-2.5	-0.1
	245	37	60	3.2	-1.6	3.1	-2.5	-2.4	0.6	2.2	0.0
	237	32	55	3.2	-1.6	-2.4	3.1	2.3	0.3	-2.2	0.0
	235	0	23	3.2	-1.6	-2.6	3.2	-2.7	0.5	2.5	0.0
	347	20	43	3.2	2.6	-1.7	-2.8	2.8	1.6	-2.1	-0.3
	234	13	36	3.2	-0.6	-2.7	-2.8	2.7	1.0	2.5	-0.5
	156	10	32	-3.2	1.8	2.8	2.8	-2.7	-0.7	2.2	0.2
	157	15	38	-3.2	2.3	2.9	2.9	-2.4	2.7	-2.3	0.2
	126	34	57	-3.2	-2.5	2.7	1.9	2.5	-1.0	2.6	-0.4
	127	32	55	-3.3	-1.4	2.8	3.1	2.3	1.9	-2.5	-0.2
	125	32	54	-3.3	-1.7	3.0	2.9	-2.5	2.3	2.2	-0.2
	136	51	73	3.2	-1.1	3.2	-2.2	-0.7	2.5	-2.2	-0.1
	137	30	52	-3.2	2.3	-2.3	3.0	2.9	2.6	-2.0	-0.4
	135	28	50	-3.2	2.4	-2.7	2.9	-1.9	2.8	2.8	-0.4
	147	26	49	-3.3	2.5	2.9	-2.7	2.9	2.8	-1.8	-0.4
	145	34	56	-3.2	2.4	3.0	-2.3	-2.0	2.7	2.8	-0.4
	123	55	78	-3.1	-2.3	-1.8	3.0	2.9	2.5	2.4	-0.8
	17	18	40	-3.2	1.7	2.7	1.6	2.0	1.3	-2.6	-0.2
T25	247	0	40	3.2	-1.5	3.2	-2.6	2.6	0.2	-2.5	0.0
	235	12	51	3.2	-2.2	-2.9	2.7	-2.6	2.2	2.6	-0.2
	346	4	44	3.3	2.6	-2.9	-2.8	2.5	-1.7	2.3	-0.3
	234	8	47	3.2	-1.8	-2.7	-2.7	2.7	2.2	2.4	-0.7
	157	16	55	-2.8	2.4	2.8	2.8	-2.6	2.5	-2.4	0.3
	147	15	55	-3.2	2.5	2.9	-2.7	2.9	2.8	-1.8	-0.4
	17	12	51	-3.2	1.8	2.7	1.7	2.0	0.9	-2.6	-0.1
T63	247	0	29	3.2	-2.4	3.0	-2.8	2.7	1.1	-1.8	-0.3
	235	2	31	3.2	-2.6	-2.8	3.0	-2.1	1.6	2.6	-0.2

	346	8	37	3.2	1.9	-3.0	-3.0	2.5	-0.6	2.4	-0.4
	157	37	66	-2.7	3.1	3.0	2.8	-2.6	1.3	-2.4	0.4
	147	27	56	-3.2	3.1	3.0	-2.7	2.8	2.0	-1.8	-0.3
T65	247	3	45	3.2	-2.5	3.1	-2.8	2.7	1.2	-1.9	-0.3
	235	0	42	3.2	-2.5	-2.8	3.0	-2.1	1.7	2.6	-0.2
	346	7	49	3.3	2.0	-3.0	-3.0	2.5	-0.7	2.3	-0.4
	347	0	42	3.2	2.8	-2.2	-2.9	2.9	1.3	-2.0	-0.3
	157	35	77	-3.3	2.9	2.9	2.9	-2.1	1.7	-1.9	0.1
	147	38	80	-2.1	2.4	2.8	-2.3	2.7	1.8	-2.2	-0.3
	17	18	60	-3.2	1.4	2.4	2.1	2.5	0.8	-2.7	-0.1
N2352	247	0	30	3.3	-2.3	3.0	-2.8	2.6	0.3	-1.4	-0.2
	237	26	56	3.3	-2.8	-2.3	3.0	2.4	1.0	-2.1	0.0
	235	11	41	3.2	-2.3	-1.9	3.1	-2.8	1.3	2.1	-0.1
	346	1	31	3.3	3.0	-2.3	-2.8	2.6	-1.2	0.2	-0.2
	127	15	45	-3.3	-2.9	2.7	2.9	2.9	2.0	-1.0	-0.3
	125	43	73	-3.3	-2.5	3.1	3.1	-2.1	2.5	2.5	-0.4
	13	23	53	-3.3	2.4	-3.1	2.6	2.8	0.7	2.0	-0.6
	14	25	55	-3.3	2.8	2.4	-3.2	2.4	2.4	0.7	-0.6
N2353	457	41	59	3.4	3.0	3.0	-2.9	-2.8	1.4	-2.6	0.3
	247	1	19	3.3	-2.4	3.0	-2.8	2.6	0.5	-1.4	-0.2
	245	10	28	3.3	-2.7	2.9	-3.0	2.7	-1.6	1.1	-0.2
	236	37	55	3.3	-2.8	-2.9	3.0	2.5	-1.9	1.5	-0.1
	237	29	47	3.3	-2.8	-2.2	3.0	2.4	0.9	-2.1	0.0
	235	56	74	-2.6	2.9	2.9	-1.2	2.6	-0.3	-1.4	-0.1
	235	12	30	3.2	-2.1	-2.1	3.1	-2.8	1.8	1.6	0.0
	346	0	18	3.3	3.0	-2.4	-2.9	2.6	-1.3	0.6	-0.3
	345	29	47	3.2	3.1	-2.0	-2.7	-2.3	1.0	2.5	-0.2
	125	45	63	-3.3	-2.5	2.9	3.0	-2.1	2.6	2.8	-0.4
	137	28	46	-3.2	2.6	-2.9	2.6	2.6	2.6	-0.9	-0.5
	135	53	71	-3.3	2.8	-2.7	3.0	-2.0	2.8	2.8	-0.4
	146	23	41	-3.3	2.7	2.9	-3.2	2.6	0.0	2.4	-0.6
	147	29	47	-3.3	2.9	2.7	-3.2	2.6	2.5	-0.4	-0.5
	145	55	73	-3.3	3.0	3.0	-2.8	-1.8	2.8	2.8	-0.5
	124	53	71	3.3	3.0	-1.9	3.2	-2.4	-1.6	-2.1	0.7
	123	68	86	-2.8	-2.7	-2.1	3.1	3.0	2.7	2.7	-1.0
	134	59	77	3.4	-1.9	3.0	3.2	-2.4	-2.0	-1.7	0.7
	12	19	37	-3.3	-3.2	2.2	2.6	2.7	2.1	0.9	-0.6
	13	28	46	-3.3	2.7	-3.0	2.8	2.6	1.7	0.5	-0.6
	14	26	44	-3.3	2.8	2.4	-3.2	2.5	2.5	0.6	-0.6
N2552	247	0	48	3.2	-2.2	3.0	-2.8	2.6	0.2	-1.4	-0.1
	235	3	51	3.2	-2.2	-2.1	3.1	-2.8	1.6	2.0	-0.1
	126	34	82	-3.3	-2.7	3.0	3.0	2.9	-1.8	2.3	-0.4
	125	4	52	3.3	3.1	-2.1	-2.5	2.8	-1.3	-1.0	0.0
	146	1	49	3.2	-2.0	-2.3	3.1	-2.8	2.0	1.6	-0.1

	147	53	101	-2.5	2.8	2.9	-1.5	2.5	0.4	-1.7	-0.1
	12	16	64	-3.3	-3.1	2.8	2.8	2.5	0.4	1.7	-0.6
	247	9	38	3.2	-2.2	3.0	-2.8	2.7	0.1	-1.4	-0.1
	235	9	39	3.2	-2.0	-2.1	3.1	-2.8	1.9	1.4	0.0
	346	0	30	3.3	3.0	-2.2	-2.9	2.7	-1.3	0.3	-0.2
	346	0	30	3.3	3.0	-2.2	-2.9	2.7	-1.3	0.3	-0.2
	126	27	56	-3.3	-3.0	2.7	3.0	2.6	-0.1	1.7	-0.5
	147	59	89	-2.5	2.8	2.9	-1.5	2.5	0.3	-1.6	-0.1
	12	25	55	-3.3	-3.0	2.7	2.9	2.5	0.1	1.8	-0.5
N2332	247	0	58	3.2	-2.4	3.1	-2.2	2.2	1.6	-2.6	-0.1
	235	5	63	3.3	-2.4	-2.8	3.0	-1.6	0.7	2.5	-0.2
	346	9	67	3.2	2.4	-3.0	-2.8	2.3	-1.6	2.6	-0.3
	347	22	80	3.3	3.1	-2.7	-2.3	2.7	0.9	-2.1	-0.2
	167	30	88	-2.9	3.0	3.0	1.7	2.2	-1.7	-2.5	0.1
	127	5	63	3.3	3.0	-2.7	-2.2	0.0	-1.1	2.5	-0.2
	146	23	81	-3.3	2.6	2.7	-3.1	2.4	-0.2	2.7	-0.5
	147	32	90	-3.4	3.1	3.0	-2.6	2.8	2.5	-2.0	-0.5
	247	1	51	3.3	-2.4	3.1	-2.2	2.4	1.3	-2.6	-0.1
	245	33	83	3.2	-2.4	3.0	-2.0	-1.9	0.5	1.9	0.1
	237	34	84	3.3	-2.3	-2.6	3.0	1.3	2.5	-2.1	-0.2
	235	9	59	3.3	-2.4	-2.8	3.0	-1.5	0.5	2.5	-0.2
	346	0	50	3.3	3.0	-2.8	-2.3	0.2	-1.2	2.5	-0.2
	347	19	68	-3.3	3.1	3.1	-2.5	-1.7	2.1	2.1	-0.2
	235	7	57	3.3	2.9	-2.9	-2.4	-1.9	1.0	2.6	-0.1
	234	29	79	3.3	-2.5	-2.8	-1.4	2.0	2.2	2.5	-0.6
	156	35	85	-3.2	2.6	3.0	1.8	-2.3	-1.3	2.5	0.1
	167	18	68	-2.9	2.9	2.9	2.9	1.9	-2.2	-2.6	0.2
	126	25	74	-3.3	-3.0	2.8	2.7	2.4	-0.6	2.4	-0.4
	127	35	85	-3.3	-2.7	3.0	2.8	2.9	2.6	-1.8	-0.4
	137	43	92	-3.3	2.9	-2.8	2.8	2.9	2.7	-1.5	-0.5
	146	23	73	-3.3	2.6	2.7	-3.1	2.5	-0.3	2.7	-0.5
	147	32	82	-3.4	3.1	3.1	-2.7	2.9	2.5	-2.0	-0.5
	145	40	90	-3.3	3.1	3.1	-2.6	-1.7	2.1	2.3	-0.2
	124	45	95	-3.4	-2.2	3.1	-2.5	2.9	2.9	2.9	-0.9
	123	60	110	3.4	3.0	3.2	-1.9	-2.4	-1.6	-2.3	0.6
	134	55	105	-3.1	3.1	-1.9	-2.7	2.9	2.9	2.9	-1.0
	12	20	70	-3.3	-3.2	2.5	2.2	1.2	2.0	2.6	-0.6
	13	12	62	-3.3	2.5	-3.2	2.5	2.8	0.2	2.5	-0.6
N2532	247	1	73	3.2	-2.4	3.1	-2.1	2.3	1.3	-2.7	-0.1
	235	6	78	3.3	-2.5	-2.9	2.8	-2.0	1.7	2.5	-0.2
	346	5	76	3.2	2.3	-3.0	-2.8	2.4	-1.5	2.6	-0.3
	347	21	92	3.3	3.2	-2.6	-2.2	2.6	0.6	-2.0	-0.1
	127	0	72	3.3	3.0	-2.7	-2.2	-0.2	-1.1	2.5	-0.1
	136	10	82	-3.2	2.6	-3.2	2.6	2.8	-0.3	2.5	-0.5
	147	34	106	-3.3	2.9	3.0	-2.7	2.8	2.7	-1.9	-0.5

	13	18	90	-3.2	2.2	-3.2	2.4	1.7	2.2	2.3	-0.7
	14	18	90	-3.3	2.4	2.7	-3.1	2.3	0.2	2.6	-0.5
	17	10	82	-3.2	2.8	2.7	1.4	1.9	0.9	-2.7	-0.2
N2535	247	8	70	3.2	-2.4	3.1	-2.1	2.2	1.4	-2.6	-0.1
	235	7	69	3.3	-2.3	-2.8	3.0	-1.5	0.5	2.5	-0.2
	346	0	62	3.3	3.0	-2.8	-2.3	0.2	-1.2	2.5	-0.2
	347	23	85	3.3	3.1	-2.6	-2.2	2.7	0.7	-2.1	-0.2
	167	27	89	-2.8	3.0	2.9	2.2	2.4	-2.3	-2.7	0.2
	127	32	93	-3.3	-2.7	3.0	2.8	2.9	2.6	-1.8	-0.4
	147	39	100	-3.3	3.1	2.9	-2.6	2.7	2.7	-2.0	-0.4
	14	25	87	-3.3	2.4	2.6	-3.1	2.4	0.1	2.6	-0.5
	17	22	84	-3.3	1.5	2.7	2.7	1.6	1.3	-2.7	-0.2
N3252	247	3	88	3.3	-2.7	3.0	-2.3	0.1	2.4	-1.2	-0.1
	235	0	85	3.3	-2.7	-2.4	3.0	-1.6	2.4	0.7	-0.2
	346	4	89	3.2	3.1	-2.4	-2.3	2.3	-2.6	1.5	-0.1
	135	21	106	3.3	-2.4	3.1	-2.1	2.7	-0.7	-1.0	-0.1
	147	23	108	-3.3	2.6	2.7	-2.8	2.7	2.6	-1.0	-0.5
	12	6	90	-3.3	-3.2	2.7	2.6	1.8	2.1	1.7	-0.7
	17	10	95	-3.3	2.7	2.8	1.3	1.8	-2.6	1.0	-0.2
N3253	247	3	78	3.2	-2.9	2.3	-2.8	2.4	2.4	-1.6	-0.3
	235	3	78	3.3	-2.7	-2.3	3.0	-1.6	2.4	0.6	-0.2
	346	0	75	3.2	3.1	-2.4	-2.3	2.3	-2.6	1.5	-0.1
	135	22	97	3.3	-2.4	3.1	-2.0	2.7	-0.7	-1.0	-0.1
	147	23	98	-3.3	2.7	2.7	-2.8	2.7	2.6	-1.2	-0.4
	12	12	87	-3.2	-3.2	2.4	2.5	0.5	2.4	2.6	-0.6
	17	7	82	-3.3	2.7	2.7	1.4	1.8	-2.7	1.2	-0.2
N3552	247	13	89	3.2	-2.9	2.2	-2.8	2.5	2.4	-1.6	-0.3
	235	6	82	3.3	-2.8	-2.5	3.0	-1.7	2.4	1.0	-0.3
	346	0	76	3.3	3.1	-2.4	-2.1	2.3	-2.6	1.4	-0.1
	234	32	108	3.2	-2.7	-1.7	-2.1	2.3	2.4	1.9	-0.7
	136	6	81	3.3	-2.7	3.0	-2.0	-0.4	2.4	-1.1	-0.1
	135	23	99	3.3	-2.4	3.1	-2.1	2.6	-0.7	-1.0	-0.1
	147	26	101	-3.3	2.6	2.6	-2.8	2.7	2.6	-1.0	-0.5
	12	7	83	-3.3	-3.2	2.7	2.4	1.7	2.1	1.9	-0.7
	17	11	87	-3.3	2.7	2.8	1.3	1.8	-2.7	1.2	-0.2
N3553	467	34	96	3.2	2.6	2.8	-2.7	1.6	-2.6	-2.4	0.4
	246	21	83	3.3	-2.7	3.1	-2.3	2.6	-2.1	1.2	-0.2
	247	17	79	3.2	-2.9	2.6	-2.7	2.1	2.5	-1.8	-0.3
	236	32	94	3.3	-2.6	-2.4	3.0	1.7	-2.2	2.5	-0.2
	235	14	76	3.3	-2.8	-2.3	3.0	-1.5	2.4	0.5	-0.2
	346	0	62	3.3	3.1	-2.4	-2.2	2.2	-2.6	1.5	-0.1
	235	26	87	3.3	2.9	-2.5	-2.1	-2.3	2.0	1.1	0.0
	234	33	94	3.2	-2.8	-1.8	-2.1	2.3	2.5	2.0	-0.7
	234	31	93	3.2	-2.8	-2.4	-1.3	2.0	2.5	2.1	-0.7

	156	16	78	-2.9	2.9	2.9	2.9	-2.4	-2.5	1.9	0.2
	167	25	87	-2.9	2.9	2.8	2.9	1.9	-2.5	-2.2	0.2
	157	32	93	-2.9	2.9	2.6	2.8	-2.6	1.1	-1.7	0.4
	146	31	92	-3.4	3.0	3.1	-2.5	2.7	-2.0	2.5	-0.4
	147	31	93	-3.3	2.7	2.6	-2.9	2.6	2.6	-0.8	-0.5
	145	36	98	-3.3	3.0	2.9	-2.7	-1.9	2.7	2.6	-0.4
	124	58	120	-3.1	-1.7	2.9	-2.7	2.9	2.9	2.8	-1.0
	123	68	129	3.5	3.1	3.0	-1.7	-2.3	-2.0	-2.1	0.7
	123	72	133	-3.0	-1.8	-2.7	3.0	3.0	2.8	2.7	-0.9
	12	14	76	-3.3	-3.2	2.4	2.4	2.3	2.2	1.7	-0.7
	13	30	91	-3.3	2.4	-3.1	2.1	1.2	2.6	2.1	-0.7
	14	26	88	-3.3	2.3	2.1	-3.1	2.4	2.6	1.1	-0.7
C2	247	9	153	3.2	-3.1	3.2	-3.2	3.2	2.8	-2.8	-0.3
	346	14	158	3.3	3.1	-3.3	-3.2	3.0	-2.5	2.9	-0.4
	345	16	160	3.4	3.0	-3.2	-3.1	-2.9	2.7	3.1	-0.4
	167	15	159	-3.3	3.0	3.1	2.9	2.8	-2.7	-3.1	0.3
	147	0	144	-3.4	3.3	3.3	-3.2	3.2	2.8	-2.9	-0.2

Table S2. Relative energies (kJ/mol) for the various BS states for the 26 structures, calculated with TPSSh (ΔE_s within the same structure, ΔE for all structures and BS states). The Mulliken spin population for all Fe ions and Mo are also listed.

Structure	BS	ΔE_S	ΔE	Fe1	Fe2	Fe3	Fe4	Fe5	Fe6	Fe7	Mo
B33	247	8	19	3.6	-3.3	3.5	-3.4	3.3	3.0	-3.3	-0.6
	235	4	14	3.6	-3.3	-3.4	3.6	-3.0	1.9	3.2	-0.2
	346	13	24	3.7	2.6	-3.4	-3.4	3.3	-2.8	3.2	-0.5
	157	0	11	-3.42	3.6	3.5	3.5	-3.3	2.0	-3.2	0.5
	135	16	30	-3.6	3.5	-3.3	3.6	-3.0	2.5	3.3	-0.4
	147	22	37	-3.6	2.9	3.6	-3.3	3.4	3.2	-2.7	-0.4
B35	247	3	10	3.6	-3.3	3.6	-3.4	3.2	1.9	-2.9	-0.3
	235	6	13	3.6	-3.4	-3.4	3.5	-3.0	2.2	3.3	-0.3
	346	14	21	3.7	2.6	-3.4	-3.4	3.2	-2.8	3.2	-0.5
	157	2	8	-3.4	3.6	3.5	3.5	-3.3	2.0	-3.2	0.5
	147	0	7	-3.6	3.5	3.6	-3.3	3.3	2.5	-2.8	-0.4
B53	247	2	26	3.6	-3.3	3.5	-3.4	3.3	2.9	-3.3	-0.6
	235	5	29	3.6	-3.1	-3.4	3.5	-3.4	2.8	3.2	-0.6
	346	0	24	3.6	2.6	-3.4	-3.4	3.2	-2.8	3.2	-0.5
	157	75	99	3.7	-2.9	-3.2	-3.2	3.7	-0.1	3.2	0.7
	147	15	39	-3.58	2.9	3.6	-3.3	3.4	3.2	-2.7	-0.5
B55	247	6	33	3.6	-3.3	3.6	-3.4	3.3	1.9	-2.9	-0.3
	235	16	42	3.6	-3.4	-3.4	3.5	-3.0	2.2	3.3	-0.3
	346	13	39	3.7	2.6	-3.4	-3.4	3.3	-2.8	3.2	-0.5
	147	0	27	-3.6	3.5	3.6	-3.3	3.3	2.6	-2.8	-0.4
H2F	247	8	79	3.6	-3.1	3.4	-3.4	3.2	3.0	-3.3	-0.6
	235	0	71	3.6	-3.1	-3.5	3.4	-3.3	3.1	3.1	-0.6
	346	3	74	3.6	3.4	-3.4	-3.4	3.1	-3.3	3.1	-0.3
	157	19	90	-3.7	2.6	3.4	3.3	-3.0	3.4	-3.0	0.2
	147	6	77	-3.6	3.1	3.6	-3.4	3.3	3.3	-2.7	-0.6
H2S	247	6	84	3.6	-3.1	3.5	-3.4	3.2	3.0	-3.2	-0.5
	235	3	81	3.61	-3.2	-3.4	3.5	-3.3	3.0	3.1	-0.6
	346	0	78	3.6	3.4	-3.4	-3.4	3.1	-3.3	3.0	-0.3
	167	36	115	-3.7	2.9	3.4	2.9	3.2	-3.1	-3.2	0.6
	157	16	95	-3.7	2.6	3.4	3.3	-3.0	3.4	-3.0	0.3
	135	12	91	-3.6	3.1	-3.4	3.5	-2.9	3.3	3.2	-0.5
	147	7	85	-3.6	3.1	3.6	-3.4	3.3	3.3	-2.7	-0.6
H6M	247	5	8	3.6	-3.3	3.5	-3.4	3.2	3.1	-3.3	-0.6
	235	0	3	3.6	-3.3	-3.4	3.5	-3.3	3.1	3.1	-0.6
	346	4	7	3.6	3.5	-3.4	-3.4	3.3	-3.5	3.2	-0.4
	234	25	28	3.7	-3.0	-3.3	-3.3	3.3	3.0	3.2	-1.6
	157	22	25	-3.7	2.9	3.4	3.4	-3.2	3.3	-3.1	0.5
	147	37	40	-3.5	3.4	3.5	-3.3	3.3	3.1	-3.1	-0.7
H6S	567	183	183	3.8	3.2	3.5	3.4	-3.5	-3.2	-3.1	-1.8
	267	118	118	3.7	-3.4	3.5	3.4	3.3	-3.3	-3.6	-1.0
	256	102	102	3.7	-3.4	3.5	3.4	-3.6	-3.3	3.2	-1.0
	257	74	74	3.4	-3.5	2.4	3.4	-3.3	3.4	-3.2	0.5
	367	80	80	3.7	3.3	-3.5	3.5	3.3	-3.5	-3.1	-1.1
	356	53	53	3.5	3.2	-3.6	3.3	-3.3	-3.6	3.1	0.7

	357	44	44	3.7	3.2	-3.6	3.5	-3.3	2.6	-3.3	0.4
	467	74	74	3.5	3.1	3.2	-3.6	3.1	-3.6	-3.3	0.7
	456	104	104	3.7	3.4	3.6	-3.4	-3.5	-3.3	3.2	-1.1
	457	64	64	3.6	3.2	3.4	-3.5	-3.3	2.6	-3.3	0.5
	246	39	39	3.6	-3.2	3.5	-3.4	3.4	-3.3	3.2	-1.3
	247	8	8	3.6	-3.3	3.5	-3.4	3.2	3.1	-3.3	-0.6
	245	28	28	3.6	-3.4	3.5	-3.5	-3.2	3.2	3.1	-0.6
	236	35	35	3.6	-3.2	-3.4	3.5	3.4	-3.2	3.3	-1.5
	237	24	24	3.6	-3.4	-3.5	3.4	3.2	3.3	-3.2	-0.6
	235	0	0	3.6	-3.3	-3.4	3.5	-3.3	3.1	3.2	-0.6
	346	8	8	3.6	3.5	-3.4	-3.4	3.3	-3.5	3.2	-0.5
	347	27	27	3.6	3.4	-3.5	-3.5	3.3	3.3	-3.1	-0.6
	345	26	26	3.6	3.4	-3.4	-3.4	-3.2	3.2	3.2	-0.6
	234	27	27	3.7	-3.0	-3.3	-3.3	3.3	3.0	3.3	-1.6
	156	31	31	-3.7	3.2	3.4	3.3	-3.2	-3.3	3.1	0.6
	157	27	27	-3.7	2.9	3.4	3.4	-3.2	3.3	-3.1	0.6
	126	48	48	-3.6	-3.3	3.5	3.5	3.3	-3.3	3.1	-0.3
	127	31	31	-3.6	-3.4	3.5	3.5	3.3	3.3	-3.2	-0.7
	125	23	23	-3.6	-3.4	3.6	3.5	-3.3	3.3	3.3	-0.7
	136	32	32	-3.6	3.3	-3.4	3.5	3.4	-3.2	3.2	-0.4
	137	45	45	-3.6	3.3	-3.4	3.5	3.3	2.9	-2.8	-0.5
	135	37	37	-3.5	3.4	-3.3	3.5	-3.2	3.3	3.3	-0.7
	146	28	28	-3.6	3.4	3.5	-3.4	3.3	-3.2	3.3	-0.4
	147	37	37	-3.6	3.4	3.5	-3.3	3.3	3.2	-3.1	-0.7
	145	45	45	-3.6	3.4	3.6	-3.3	-3.0	2.9	3.3	-0.5
	124	61	61	-3.7	-3.1	3.6	-3.4	3.5	3.7	3.4	-1.6
	123	80	80	-3.6	-3.1	-3.4	3.6	3.5	3.7	3.4	-1.6
	134	84	84	-3.7	3.6	-3.3	-3.2	3.6	3.7	3.5	-1.6
D26	247	5	50	3.6	-2.9	3.4	-3.4	3.2	2.5	-3.2	-0.4
	235	0	45	3.6	-2.9	-3.4	3.4	-3.3	2.5	3.2	-0.5
	346	13	59	3.6	3.1	-3.4	-3.4	3.2	-2.9	3.1	-0.5
	234	10	55	3.7	-2.6	-3.2	-3.2	3.3	2.4	3.2	-1.5
	156	21	66	-3.7	2.6	3.3	3.2	-3.2	-2.4	3.0	0.4
	156	74	112	-3.4	2.9	2.5	3.3	-3.3	-2.5	2.8	0.5
	147	42	87	-3.6	3.0	3.6	-3.3	3.3	2.6	-2.7	-0.4
T23	247	12	43	3.6	-2.9	3.5	-3.5	3.2	2.7	-3.3	-0.6
	235	5	37	3.6	-2.8	-3.5	3.5	-3.3	2.7	3.1	-0.6
	346	0	31	3.6	3.0	-3.4	-3.4	3.2	-2.9	3.1	-0.4
	234	22	53	3.7	-2.6	-3.3	-3.3	3.3	2.8	3.2	-1.5
	147	4	35	-3.6	3.0	3.6	-3.3	3.3	3.2	-2.8	-0.5
T25	247	10	52	3.6	-2.9	3.5	-3.4	3.2	2.7	-3.3	-0.6
	235	6	48	3.6	-2.9	-3.5	3.3	-3.3	3.1	3.1	-0.5
	346	0	42	3.6	3.1	-3.4	-3.4	3.2	-2.9	3.1	-0.5
	234	18	60	3.7	-2.6	-3.3	-3.3	3.3	2.9	3.2	-1.5
	157	13	55	-3.4	3.0	3.5	3.5	-3.3	2.6	-3.2	0.5
	147	1	42	-3.6	2.9	3.6	-3.3	3.3	3.2	-2.8	-0.5
T63	247	4	34	3.6	-3.4	3.5	-3.4	3.3	2.4	-2.9	-0.3
	235	0	30	3.6	-3.4	-3.4	3.5	-3.1	2.4	3.2	-0.2
	346	24	54	3.6	3.1	-3.4	-3.4	3.2	-2.6	3.1	-0.6
	157	36	66	-3.4	3.4	3.4	3.3	-3.3	2.4	-3.3	0.7

	135	25	55	-3.5	3.5	-3.3	3.5	-3.0	2.5	3.3	-0.3
	147	20	50	-3.6	3.5	3.5	-3.3	3.3	2.5	-2.8	-0.4
T65	247	2	44	3.6	-3.4	3.5	-3.4	3.3	2.4	-2.9	-0.3
	235	0	41	3.6	-3.4	-3.4	3.5	-3.1	2.4	3.2	-0.2
	346	33	74	3.6	3.0	-3.4	-3.4	3.2	-2.7	3.1	-0.6
	157	37	78	-3.7	3.4	3.5	3.4	-3.0	2.1	-2.8	0.7
	135	23	64	-3.5	3.5	-3.3	3.5	-3.0	2.5	3.3	-0.3
	147	80	121	-2.8	3.3	3.4	-2.9	3.3	2.2	-3.2	-0.5
N2352	247	0	21	3.6	-3.4	3.3	-3.5	3.2	2.9	-3.0	-0.3
	235	14	36	3.6	-3.3	-3.1	3.5	-3.3	2.7	3.0	-0.6
	346	15	37	3.6	3.6	-3.2	-3.4	3.3	-3.2	3.3	-1.5
	125	9	30	-3.6	-3.3	3.5	3.6	-3.0	3.2	3.0	-0.5
	147	18	40	-3.6	3.5	3.5	-3.4	3.3	3.0	-2.7	-0.6
N2353	247	7	21	3.6	-3.3	3.5	-3.4	3.2	2.6	-2.9	-0.4
	235	16	30	3.6	-3.4	-3.4	3.5	-3.0	2.7	3.1	-0.5
	346	0	14	3.6	3.3	-3.4	-3.5	3.2	-2.9	3.0	-0.4
	125	62	77	3.6	3.5	-2.9	-3.3	3.6	-1.8	-2.6	1.9
	147	66	80	3.6	-2.7	-2.5	3.6	-3.2	-2.2	3.5	1.7
N2552	247	6	45	3.6	-3.3	3.5	-3.4	3.2	2.6	-2.9	-0.5
	235	104	142	-3.3	3.4	3.4	-1.4	3.3	-1.3	-2.4	0.4
	235	7	45	3.6	-3.2	-3.2	3.5	-3.3	3.0	2.8	-0.6
	346	0	39	3.6	3.4	-3.4	-3.4	3.2	-2.8	2.7	-0.7
	125	40	79	3.6	3.5	-2.7	-3.0	3.4	-1.8	-1.9	0.5
	147	7	46	-3.6	3.4	3.4	-3.5	3.3	3.2	-2.7	-0.4
N2553	247	0	22	3.6	-3.4	3.3	-3.5	3.2	2.9	-3.0	-0.3
	235	106	128	-3.3	3.4	3.4	-1.4	3.3	-1.4	-2.3	0.4
	346	8	30	3.6	3.5	-3.3	-3.4	3.2	-2.8	2.6	-0.7
	125	60	82	3.6	3.4	-3.0	-3.4	3.6	-1.8	-2.6	1.9
	147	71	93	3.6	-2.8	-2.9	3.6	-3.2	-1.5	3.4	1.7
N2332	247	18	50	3.6	-3.2	3.5	-3.1	3.1	2.6	-3.3	-0.5
	235	6	38	3.6	-3.4	-3.5	3.3	-3.0	3.0	3.2	-0.4
	346	0	32	3.5	3.3	-3.5	-3.4	3.1	-2.8	3.2	-0.3
	127	32	64	-3.6	-3.4	3.4	3.3	3.1	3.4	-3.3	0.6
	147	8	40	-3.7	3.5	3.6	-3.3	3.2	3.0	-2.9	-0.6
N2335	247	11	39	3.6	-3.3	3.6	-3.1	3.1	2.6	-3.3	-0.5
	235	0	28	3.6	-3.4	-3.5	3.2	-2.9	3.1	3.2	-0.5
	346	9	37	3.6	3.4	-3.5	-3.4	3.0	-3.0	3.1	-0.3
	127	63	90	3.6	3.6	-2.9	-2.7	-2.1	-1.8	3.3	0.5
	147	2	30	-3.7	3.5	3.6	-3.3	3.3	3.0	-2.9	-0.6
N2532	247	20	65	3.6	-3.2	3.5	-3.1	3.1	2.6	-3.3	-0.5
	235	0	45	3.6	-3.4	-3.5	3.2	-3.0	3.1	3.2	-0.3
	346	8	53	3.6	3.2	-3.5	-3.4	3.1	-2.8	3.1	-0.4
	127	80	125	3.6	3.5	-3.3	-2.9	-2.8	-1.8	3.6	1.9
	147	32	77	-3.6	3.5	3.6	-3.1	3.2	2.9	-3.1	-0.6
	13	72	117	-3.6	2.7	-3.6	2.9	2.1	2.4	2.8	-1.7
N2535	247	9	57	3.6	-3.3	3.5	-3.1	3.1	2.6	-3.3	-0.5
	235	18	66	3.6	-3.4	-3.4	3.4	-2.9	2.7	3.2	-0.7
	346	0	47	3.6	3.4	-3.5	-3.4	3.0	-3.0	3.1	-0.3
	127	54	101	3.6	3.6	-2.9	-2.7	-2.1	-1.8	3.3	0.6

	147	13	60	-3.6	3.4	3.5	-3.3	3.3	3.1	-2.9	-0.5
N3252	247	12	94	3.6	-3.4	3.4	-3.3	2.8	3.1	-2.9	-0.5
	235	2	85	3.6	-3.4	-3.3	3.4	-2.9	3.2	2.7	-0.6
	346	0	82	3.6	3.5	-3.3	-3.1	3.1	-3.3	2.7	-0.5
	125	87	134	3.6	3.6	-2.6	-2.5	3.5	-3.0	-2.4	1.7
	147	44	91	-3.6	3.4	3.4	-3.3	3.2	3.1	-2.8	-0.5
N3253	247	0	66	3.5	-3.4	3.2	-3.4	3.1	3.1	-2.9	-0.4
	247	3	69	3.6	-3.4	3.3	-3.4	3.1	3.1	-3.0	-0.3
	235	8	75	3.6	-3.4	-3.4	3.4	-2.9	3.2	2.8	-0.7
	346	2	68	3.6	3.5	-3.2	-3.2	3.0	-3.2	2.8	-0.5
	125	56	123	3.6	3.6	-2.6	-2.5	3.5	-3.0	-2.4	1.7
	147	16	83	-3.6	3.4	3.3	-3.3	3.2	3.1	-2.8	-0.5
N3552	247	0	65	3.6	-3.4	3.3	-3.4	3.1	3.1	-2.9	-0.3
	235	14	79	3.6	-3.4	-3.3	3.4	-2.9	3.2	2.7	-0.7
	346	-2	63	3.6	3.6	-3.3	-3.1	3.3	-3.4	3.2	-1.4
	125	59	126	3.6	3.6	-2.6	-2.5	3.5	-3.0	-2.3	1.6
	125	61	126	3.6	3.6	-2.6	-2.5	3.5	-3.0	-2.3	1.6
	147	14	81	-3.6	3.5	3.3	-3.3	3.3	3.1	-2.9	-0.5
N3553	247	97	153	-3.4	3.6	0.0	3.6	-3.1	-3.2	3.4	1.6
	235	15	71	3.6	-3.4	-3.3	3.4	-2.9	3.2	2.8	-0.8
	346	0	56	3.6	3.5	-3.2	-3.1	3.0	-3.3	2.7	-0.5
	125	61	117	3.6	3.6	-2.7	-2.5	3.5	-3.0	-2.3	1.6
	147	21	77	-3.6	3.4	3.3	-3.3	3.2	3.1	-2.8	-0.5
C2	247	30	58	3.6	-3.5	3.6	-3.5	3.3	3.2	-3.4	-0.3
	235	22	50	3.7	-3.6	-3.7	3.6	-3.2	3.2	3.4	-0.6
	346	23	51	3.6	3.7	-3.6	-3.6	3.3	-3.2	3.4	-0.7
	345	18	46	3.6	3.5	-3.6	-3.6	-3.3	3.3	3.4	-0.7
	167	56	84	-3.7	3.6	3.6	3.5	3.4	-3.6	-3.3	-0.2
	147	0	28	-3.7	3.6	3.6	-3.6	3.5	3.3	-3.3	-0.4

Table S3. Relative energies (kJ/mol) for the various BS states for the 26 structures, calculated with r^2SCAN (ΔE_s within the same structure, ΔE for all structures and BS states). The Mulliken spin population for all Fe ions and Mo are also listed.

Structure	BS	ΔE_S	ΔE	Fe1	Fe2	Fe3	Fe4	Fe5	Fe6	Fe7	Mo
B33	567	81	113	3.0	2.0	3.3	3.3	-3.4	-3.2	-3.3	1.7
	267	70	102	3.5	-3.4	3.3	3.2	3.1	-2.9	-3.3	-0.6
	257	25	57	3.5	-3.5	3.5	3.5	-3.2	1.1	-3.1	0.9
	367	28	60	3.5	2.6	-3.5	3.3	3.2	-3.2	-3.2	0.5
	356	9	41	3.5	2.5	-3.6	3.4	-3.3	-3.2	3.1	0.6
	357	1	33	3.6	3.2	-3.5	3.5	-3.3	2.4	-3.1	0.5
	467	21	53	3.6	2.0	3.4	-3.5	3.3	-3.1	-3.2	0.6
	456	37	69	3.6	2.4	3.4	-3.5	-3.2	-3.2	3.1	0.5
	457	17	49	3.6	3.2	3.5	-3.5	-3.2	2.4	-3.2	0.5
	246	14	45	3.6	-3.4	3.4	-3.5	3.4	-2.8	3.3	-1.7
	247	0	32	3.6	-3.3	3.4	-3.4	3.2	2.9	-3.2	-0.6
	245	38	69	3.6	-3.4	3.5	-3.1	-3.1	2.2	3.2	-0.4
	236	8	39	3.6	-3.3	-3.5	3.4	3.3	-2.8	3.3	-1.8
	237	17	48	3.6	-3.4	-3.4	3.4	3.3	2.9	-3.1	-0.6
	235	9	41	3.6	-3.2	-3.3	3.5	-2.9	1.9	3.2	-0.3
	346	10	42	3.6	3.3	-3.4	-3.4	3.1	-3.1	3.1	-0.4
	347	15	46	3.6	3.3	-3.4	-3.3	3.3	2.8	-2.9	-0.5
	345	2	33	3.6	3.3	-3.4	-3.0	-3.0	2.4	3.3	-0.5
	234	30	62	3.7	-3.2	-3.3	-3.2	3.4	3.2	3.4	-1.7
	156	25	56	-3.7	2.7	3.4	3.4	-3.2	-3.0	3.2	0.6
	167	25	57	-3.7	3.0	3.3	3.3	3.2	-3.0	-3.2	0.7
	157	23	54	-3.3	3.6	3.4	3.4	-3.3	1.8	-3.2	0.6
	126	24	56	-3.6	-3.2	3.2	3.1	3.2	-2.4	3.1	-0.7
	127	21	53	-3.6	-3.3	3.4	3.5	3.4	3.0	-3.0	-0.6
	125	31	63	-3.6	-3.2	3.5	3.5	-3.2	3.0	3.2	-0.6
	136	17	49	-3.6	3.1	-3.5	3.0	3.1	-0.9	3.0	-1.0
	137	25	57	-3.6	3.4	-3.2	3.6	3.4	2.8	-2.8	-0.6
	135	23	55	-3.6	3.4	-3.2	3.6	-2.8	2.7	3.3	-0.5
	146	31	62	-3.6	3.4	3.5	-3.4	3.2	-2.8	3.2	-0.5
	147	19	50	-3.6	3.5	3.6	-3.2	3.4	2.6	-2.7	-0.6
	145	21	53	-3.6	3.5	3.6	-3.1	-2.8	2.6	3.3	-0.6
	124	28	59	-3.7	-2.1	3.5	-3.4	3.4	3.3	3.4	-1.7
	123	22	54	-3.6	-2.3	-3.4	3.5	3.5	3.2	3.4	-1.7
	134	83	115	-3.6	3.5	-3.3	-3.2	3.7	2.4	3.3	0.2
B35	247	16	32	3.6	-3.3	3.5	-3.3	3.2	1.9	-2.8	-0.3
	235	0	16	3.6	-3.3	-3.4	3.4	-3.3	3.0	3.1	-0.6
	157	33	49	-3.3	3.5	3.4	3.5	-3.3	1.9	-3.2	0.6
	147	20	37	-3.6	3.5	3.6	-3.2	3.3	2.6	-2.8	-0.5
B53	247	0	34	3.6	-3.2	3.4	-3.4	3.2	2.9	-3.2	-0.6
	235	6	40	3.6	-3.1	-3.4	3.5	-3.3	2.8	3.1	-0.6
	346	9	43	3.6	3.2	-3.4	-3.4	3.2	-3.1	3.1	-0.4
	157	9	43	-3.7	2.9	3.3	3.3	-3.3	3.0	-3.3	1.4
	147	24	59	-3.6	3.0	3.5	-3.3	3.3	3.1	-2.6	-0.6
B55	247	16	56	3.6	-3.3	3.5	-3.3	3.2	1.9	-2.8	-0.4
	235	0	40	3.6	-3.3	-3.4	3.4	-3.3	3.0	3.2	-0.6

	346	13	52	3.6	3.3	-3.4	-3.4	3.2	-3.2	3.1	-0.4
	157	30	69	-3.3	3.5	3.4	3.5	-3.3	1.9	-3.2	0.6
	147	16	56	-3.6	3.4	3.6	-3.2	3.3	2.7	-2.7	-0.5
H2F	247	6	85	3.6	-3.2	3.4	-3.4	3.2	3.0	-3.2	-0.5
	235	1	79	3.6	-3.2	-3.4	3.4	-3.3	3.0	3.1	-0.6
	346	0	79	3.6	3.4	-3.4	-3.4	3.1	-3.3	3.0	-0.3
	157	18	96	-3.7	2.7	3.3	3.3	-3.0	3.3	-2.9	0.2
	147	9	88	-3.6	3.2	3.5	-3.4	3.2	3.3	-2.7	-0.7
H2S	247	6	89	3.6	-3.2	3.4	-3.4	3.2	2.9	-3.2	-0.5
	235	5	87	3.6	-3.2	-3.4	3.4	-3.3	3.0	3.1	-0.5
	346	0	82	3.6	3.4	-3.4	-3.4	3.1	-3.2	3.0	-0.4
	157	22	104	-3.7	2.7	3.4	3.3	-3.0	3.3	-3.0	0.3
	135	19	101	-3.6	3.3	-3.4	3.5	-2.8	3.3	3.2	-0.6
	147	14	96	-3.6	3.3	3.5	-3.4	3.2	3.2	-2.7	-0.6
H6M	247	6	7	3.6	-3.3	3.4	-3.4	3.1	3.2	-3.3	-0.6
	235	0	1	3.6	-3.3	-3.4	3.4	-3.3	3.2	3.1	-0.6
	346	4	5	3.6	3.4	-3.4	-3.4	3.2	-3.5	3.2	-0.5
	234	20	21	3.6	-3.0	-3.3	-3.2	3.3	3.1	3.2	-1.7
	157	14	15	-3.7	2.9	3.3	3.3	-3.2	3.3	-3.2	0.8
	147	34	35	-3.5	3.4	3.5	-3.3	3.3	3.4	-3.1	-0.8
H6S	247	7	7	3.6	-3.3	3.4	-3.4	3.2	3.2	-3.3	-0.6
	235	0	0	3.6	-3.3	-3.4	3.4	-3.3	3.2	3.1	-0.7
	346	6	6	3.6	3.4	-3.4	-3.4	3.3	-3.5	3.2	-0.5
	234	22	22	3.7	-3.0	-3.3	-3.2	3.3	3.1	3.2	-1.7
	157	17	17	-3.6	2.9	3.3	3.3	-3.3	3.3	-3.2	0.8
	147	34	34	-3.5	3.4	3.5	-3.3	3.3	3.4	-3.1	-0.7
D26	247	4	49	3.6	-2.9	3.4	-3.4	3.2	2.6	-3.2	-0.4
	235	0	44	3.6	-2.9	-3.4	3.4	-3.2	2.6	3.1	-0.5
	346	16	60	3.6	3.0	-3.3	-3.3	3.2	-2.9	3.1	-0.6
	234	12	56	3.6	-2.6	-3.1	-3.1	3.3	2.5	3.2	-1.6
	156	18	62	-3.6	2.6	3.3	3.2	-3.1	-2.5	3.0	0.5
	147	45	89	-3.6	3.0	3.6	-3.2	3.3	2.6	-2.5	-0.5
T23	247	17	60	3.5	-3.0	3.4	-3.4	3.2	2.9	-3.3	-0.4
	235	7	50	3.6	-2.9	-3.4	3.4	-3.3	2.8	3.2	-0.5
	346	2	45	3.6	3.1	-3.4	-3.4	3.2	-2.7	3.1	-0.5
	157	0	43	-3.7	2.6	3.3	3.3	-3.1	3.3	-3.1	0.7
	147	4	47	-3.6	3.0	3.5	-3.3	3.3	3.2	-2.7	-0.5
T25	247	16	66	3.5	-3.0	3.4	-3.4	3.2	2.9	-3.3	-0.5
	235	12	62	3.6	-3.0	-3.4	3.3	-3.3	3.0	3.1	-0.5
	346	4	54	3.6	3.1	-3.4	-3.4	3.1	-2.8	3.1	-0.5
	157	0	51	-3.7	2.6	3.3	3.3	-3.2	3.3	-3.1	0.9
	147	3	53	-3.6	3.0	3.5	-3.3	3.3	3.2	-2.7	-0.5
T63	247	2	51	3.6	-3.3	3.5	-3.3	3.2	2.4	-2.8	-0.4
	235	0	48	3.6	-3.3	-3.3	3.5	-3.0	2.4	3.2	-0.3
	346	23	71	3.6	2.9	-3.5	-3.5	3.1	-1.8	3.0	-0.8
	157	33	81	-3.3	3.4	3.5	3.4	-3.3	1.6	-3.2	0.9
	135	20	69	-3.5	3.5	-3.2	3.4	-2.9	2.5	3.3	-0.4
	147	15	64	-3.6	3.5	3.5	-3.2	3.3	2.5	-2.7	-0.5
T65	247	2	60	3.6	-3.3	3.4	-3.3	3.2	2.4	-2.8	-0.4

	235	0	58	3.6	-3.3	-3.3	3.4	-3.0	2.5	3.2	-0.3
	346	22	80	3.6	2.9	-3.5	-3.5	3.1	-1.8	3.0	-0.8
	157	30	89	-3.2	3.4	3.4	3.5	-3.3	1.6	-3.2	1.0
	135	18	76	-3.5	3.5	-3.2	3.4	-2.9	2.5	3.3	-0.3
	147	14	72	-3.6	3.5	3.5	-3.2	3.3	2.5	-2.7	-0.5
N2352	247	0	53	3.6	-3.4	3.2	-3.4	3.2	2.9	-2.9	-0.3
	235	13	65	3.6	-3.2	-3.0	3.5	-3.3	2.6	3.0	-0.6
	346	0	53	3.6	3.5	-3.1	-3.4	3.3	-3.2	3.3	-1.5
	125	3	56	-3.6	-3.3	3.3	3.5	-3.2	3.3	3.1	-0.1
	147	6	59	-3.6	3.4	3.4	-3.4	3.3	3.1	-2.5	-0.6
N2353	247	2	49	3.6	-3.3	3.4	-3.4	3.2	2.5	-2.8	-0.5
	235	17	63	3.6	-3.3	-3.2	3.5	-3.1	2.6	3.0	-0.5
	346	3	49	3.6	3.4	-3.3	-3.4	3.2	-2.8	2.6	-0.5
	125	5	52	-3.6	-3.2	3.4	3.5	-3.0	3.3	3.1	-0.7
	147	0	47	-3.6	3.4	3.4	-3.4	3.3	3.1	-2.5	-0.6
N2552	247	1	60	3.6	-3.4	3.2	-3.4	3.2	2.9	-2.9	-0.3
	235	18	78	3.6	-3.3	-3.3	3.5	-3.1	3.0	2.7	-0.6
	346	0	59	3.6	3.5	-3.1	-3.4	3.3	-3.2	3.2	-1.5
	125	1	60	-3.6	-3.4	3.3	3.4	-3.3	3.3	3.0	0.2
	146	14	73	-3.6	3.4	3.4	-3.4	3.3	-2.4	3.1	-0.7
	147	11	70	-3.6	3.3	3.4	-3.4	3.3	3.2	-2.5	-0.5
N2553	247	3	52	3.6	-3.4	3.2	-3.4	3.2	2.9	-2.8	-0.4
	235	16	65	3.6	-3.3	-3.3	3.5	-3.0	3.1	2.7	-0.6
	346	0	49	3.6	3.5	-3.0	-3.4	3.3	-3.3	3.2	-1.5
	125	8	57	-3.6	-3.2	3.4	3.6	-3.0	3.3	3.0	-0.7
	147	8	57	-3.6	3.3	3.4	-3.4	3.3	3.2	-2.4	-0.6
N2332	247	11	79	3.6	-3.2	3.5	-3.0	3.0	2.5	-3.3	-0.5
	235	0	69	3.6	-3.4	-3.4	3.2	-2.9	2.9	3.2	-0.4
	346	4	73	3.6	3.5	-3.4	-3.1	3.3	-3.3	3.3	-1.5
	127	10	78	-3.6	-3.4	3.4	3.3	3.1	3.3	-3.3	0.4
	135	16	85	-3.6	3.3	-3.4	3.4	-2.6	3.2	3.2	-0.6
	147	2	70	-3.6	3.4	3.6	-3.2	3.2	3.0	-2.8	-0.7
N2335	247	9	69	3.6	-3.2	3.5	-3.0	3.1	2.5	-3.3	-0.5
	235	6	66	3.6	-3.4	-3.4	3.2	-2.8	2.9	3.2	-0.5
	346	9	69	3.5	3.2	-3.5	-3.4	3.0	-2.8	3.1	-0.3
	127	6	66	-3.6	3.4	3.6	-3.2	3.2	3.0	-2.8	-0.7
	147	0	60	-3.6	-3.4	3.4	3.2	3.1	3.3	-3.3	0.4
N2532	247	18	94	3.2	-3.5	3.4	-3.4	3.1	3.3	-3.4	0.6
	235	0	76	3.6	-3.4	-3.4	3.2	-2.9	2.9	3.2	-0.4
	346	15	91	3.3	3.4	-3.4	-3.4	3.1	-2.7	3.1	-0.5
	127	2	78	-3.6	-3.4	3.4	3.3	3.1	3.3	-3.3	0.4
	135	18	94	-3.6	3.3	-3.4	3.4	-2.6	3.2	3.2	-0.6
	147	10	87	-3.6	3.3	3.5	-3.2	3.2	3.1	-2.9	-0.5
	13	57	133	-3.5	2.7	-3.6	2.8	2.2	2.4	2.8	-1.7
N2535	247	19	88	3.2	-3.4	3.5	-3.4	3.2	3.2	-3.0	-0.1
	235	5	74	3.5	-3.3	-3.4	3.1	-2.8	3.0	3.2	-0.5
	346	0	69	3.6	3.5	-3.4	-3.0	3.3	-3.4	3.3	-1.6
	127	0	69	-3.6	-3.4	3.4	3.2	3.1	3.3	-3.3	0.4
	147	9	78	-3.6	3.4	3.5	-3.2	3.2	3.1	-2.9	-0.6

N3252	247	14	110	3.6	-3.4	3.2	-3.4	3.0	3.1	-2.9	-0.4
	235	16	112	3.6	-3.4	-3.3	3.3	-2.9	3.2	2.7	-0.6
	346	0	96	3.6	3.5	-3.2	-3.0	3.2	-3.4	3.2	-1.4
	125	8	105	-3.6	-3.4	3.4	3.4	-2.6	3.2	3.1	-0.7
	147	21	118	-3.6	3.3	3.3	-3.3	3.2	3.1	-2.7	-0.6
N3253	247	18	103	3.6	-3.4	3.3	-3.4	3.0	3.1	-2.8	-0.5
	235	19	104	3.6	-3.4	-3.3	3.4	-2.9	3.2	2.7	-0.7
	346	0	85	3.6	3.5	-3.1	-3.1	3.2	-3.4	3.2	-1.4
	125	6	90	-3.6	-3.4	3.3	3.4	-2.6	3.2	3.2	-0.7
	147	22	107	-3.6	3.4	3.3	-3.3	3.2	3.1	-2.6	-0.6
N3552	247	11	97	3.6	-3.4	3.2	-3.4	3.0	3.1	-2.9	-0.4
	235	20	107	3.6	-3.4	-3.3	3.4	-2.9	3.2	2.7	-0.7
	346	0	87	3.6	3.5	-3.2	-3.0	3.2	-3.4	3.2	-1.4
	125	16	103	-3.6	-3.4	3.4	3.4	-2.5	3.2	3.1	-0.8
	147	24	111	-3.6	3.4	3.3	-3.3	3.2	3.1	-2.7	-0.5
N3553	247	16	91	3.6	-3.4	3.2	-3.4	2.9	3.1	-2.8	-0.5
	235	23	98	3.6	-3.4	-3.2	3.4	-2.9	3.2	2.7	-0.8
	346	0	76	3.6	3.5	-3.1	-3.1	3.2	-3.4	3.2	-1.4
	125	14	90	-3.6	-3.4	3.3	3.4	-2.6	3.2	3.2	-0.7
	147	25	101	-3.6	3.4	3.3	-3.3	3.1	3.1	-2.6	-0.6
C2	247	11	119	3.5	-3.5	3.5	-3.5	3.3	3.3	-3.4	-0.3
	235	24	132	3.6	-3.6	-3.6	3.5	-3.2	3.2	3.4	-0.7
	346	26	134	3.6	3.6	-3.6	-3.5	3.3	-3.2	3.4	-0.8
	345	2	110	3.6	3.5	-3.5	-3.5	-3.3	3.3	3.4	-0.8
	167	30	138	-3.7	3.4	3.4	3.3	3.2	-3.4	-3.4	0.7
	147	0	108	-3.6	3.5	3.6	-3.5	3.5	3.2	-3.4	-0.4

Table S4. Relative energies (kJ/mol) of the eight best structures, QM/MM-optimised with TPSSh or r^2SCAN (and def2-SV(P) basis set) with the surrounding protein and water (within 6 Å of the QM system) allowed to relax by MM. The calculations were obtained with the BS specified in Table 2 for each method.

Structure	TPSSh	r^2SCAN
B33	20.4	39.1
B35	26.1	49.0
B53	25.2	37.3
H6M	0.0	1.1
H6S	1.7	0.0
D26	51.9	48.3
N2353	7.9	54.5
C2	33.4	111.4
